# ISPD produces CDP-ribitol used by FKTN and FKRP to transfer ribitol phosphate onto α-dystroglycan

**DOI:** 10.1038/ncomms11534

**Published:** 2016-05-19

**Authors:** Isabelle Gerin, Benoît Ury, Isabelle Breloy, Céline Bouchet-Seraphin, Jennifer Bolsée, Mathias Halbout, Julie Graff, Didier Vertommen, Giulio G. Muccioli, Nathalie Seta, Jean-Marie Cuisset, Ivana Dabaj, Susana Quijano-Roy, Ammi Grahn, Emile Van Schaftingen, Guido T. Bommer

**Affiliations:** 1 WELBIO and de Duve Institute, Biological Chemistry, Université Catholique de Louvain, B-1200 Brussels, Belgium; 2 Institute for Biochemistry II, Medical Faculty, University of Cologne, D-50931 Cologne, Germany; 3 AP-HP, Hôpital Bichat-Claude Bernard, Laboratoire de Biochimie Métabolique et Cellulaire, F-75018 Paris, France; 4 Louvain Drug Research Institute, Université Catholique de Louvain, B-1200 Brussels, Belgium; 5 Hôpital Roger-Salengro, Service de neuropédiatrie, Centre de Référence des Maladies Neuromusculaires, CHRU, F-59000 Lille, France; 6 AP-HP, Hôpital R Poincaré, Service de pédiatrie, F-92380 Garches, France; 7 Centre de Référence des Maladies Neuromusculaires, F-92380 Garches, France; 8 Université de Versailles-St Quentin, U1179 UVSQ - INSERM, F-78180 Montigny, France

## Abstract

Mutations in genes required for the glycosylation of α-dystroglycan lead to muscle and brain diseases known as dystroglycanopathies. However, the precise structure and biogenesis of the assembled glycan are not completely understood. Here we report that three enzymes mutated in dystroglycanopathies can collaborate to attach ribitol phosphate onto α-dystroglycan. Specifically, we demonstrate that isoprenoid synthase domain-containing protein (ISPD) synthesizes CDP-ribitol, present in muscle, and that both recombinant fukutin (FKTN) and fukutin-related protein (FKRP) can transfer a ribitol phosphate group from CDP-ribitol to α-dystroglycan. We also show that ISPD and FKTN are essential for the incorporation of ribitol into α-dystroglycan in HEK293 cells. Glycosylation of α-dystroglycan in fibroblasts from patients with hypomorphic ISPD mutations is reduced. We observe that in some cases glycosylation can be partially restored by addition of ribitol to the culture medium, suggesting that dietary supplementation with ribitol should be evaluated as a therapy for patients with ISPD mutations.

## Abstract (web summary)

Mutations in genes required for the glycosylation of α-dystroglycan lead to dystroglycanopathies. Here, the authors show that three of these enzymes (ISPD, FKTN and FKRP) work together to attach ribitol phosphate to α-dystroglycan.

## Graphical abstract

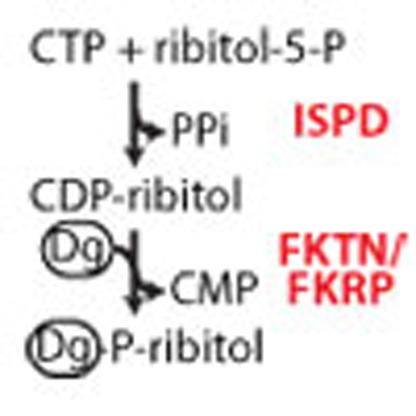

Defects in the glycosylation of α-dystroglycan are observed in a group of congenital syndromes characterized to a varying degree by muscle, brain and/or eye disorders. This group of syndromes is commonly referred to as dystroglycanopathies^1^,^2^. The clinical spectrum is vast. It ranges from limb girdle muscular dystrophy occurring after 6 months of age and lacking central nervous system symptoms, to congenital muscular dystrophy with severe brain and eye involvement (for example, muscle-eye-brain disease, Walker-Warburg syndrome and Fukuyama congenital muscle dystrophy)^1^,^3^. Reflecting the complex series of events required to obtain the glycosylation pattern necessary for α-dystroglycan function, mutations in many different genes lead to dystroglycanopathies^1^,^4^. The characterization of the affected genes has considerably expanded our knowledge not only of α-dystroglycan glycosylation, but also of the biogenesis of *O*-mannosylated proteins in general.

The Dystrophin-associated Glycoprotein 1 (*DAG1*) gene initially gives rise to a single protein product that is cleaved into an N-terminal extracellular part, α-dystroglycan and a C-terminal part, the transmembrane protein β-dystroglycan. The intracellular part of β-dystroglycan is anchored to the actin cytoskeleton via interaction with dystrophin^5^. α-Dystroglycan binds to the extracellular part of β-dystroglycan as well as to components of the extracellular matrix such as laminin, agrin and perlecan^5^. It is the interaction with laminin that is compromised by defects in glycosylation of α-dystroglycan^2^. The C-terminal part of α-dystroglycan contains mainly mucin-type *O*-glycans, while the N-terminal part contains several glycans dependent on *O*-mannosylation^6^,^7^. Mutations of genes implicated in *O*-mannosylation, as well as in the production of *O*-mannosyl glycans lead to dystroglycanopathies^4^. The genes include dolichol kinase (DOLK) involved in the formation of dolichol phosphate; dolichol phosphate mannosyl transferase polypeptide 1, 2 and 3 (DPM1, DPM2 and DPM3) implicated in the production of dolichol phosphomannose; protein *O*-mannosyltransferase 1 and 2 (POMT1 and POMT2) implicated in the transfer of mannose to specific serine or threonine residues; GDP-mannose pyrophosphorylase involved in GDP-mannose synthesis^8^; and protein *O*-linked mannose β-1,2-N-acetylglucosaminyltransferase (reviewed in (ref. 1)). In addition, several genes mutated in dystroglycanopathies contribute to the formation of a phosphorylated *O*-mannosyl-glycan, which is produced by consecutive addition of β-1,4-N-acetylglucosamine via GTDC2/AGO61 (refs 9, 10), of β-1,3-N-acetylgalactosamine via B3GALNT2 (refs 11, 12) and a phosphate group to carbon 6 of mannose via POMK1/SGK196 (ref. 11). In mature α-dystroglycan between four and seven phosphate groups seem to be present as phosphodiesters^13^,^14^. Protected from the attack by phosphatases by an unknown structure, this scaffold is modified by a chain of many repetitions of a [-3-xylose-α1,3-glucuronic acid-β1-]-disaccharide. This terminal modification is initiated by B4GAT1 (refs 15, 16) and finished by the enzymes LARGE or LARGE2, which have both xylosyltransferase and glucuronosyltransferase activity^13^,^17^.

At present, the molecular functions of several genes mutated in dystroglycanopathies are not known. This includes *ISPD*^18^,^19^,^20^,^21^, fukutin (*FKTN*)^2^,^22^, fukutin-related protein (*FKRP*)^23^,^24^, *TMEM5* (refs 15, 19) and *SLC35A1* (ref. 25). In the present paper, we are presenting evidence linking biochemical activities of ISPD, FKTN and FKRP to α-dystroglycan glycosylation as previously suggested based on the synergistic effect of morpholinos targeting these genes in zebrafish^18^.

ISPD is predicted to be a cytoplasmic protein^26^. Orthologs of ISPD are implicated in two biochemical reactions. On the one hand, they are implicated in the formation of isopentenyl pyrophosphate, a central intermediate in the biosynthesis of isoprenoids, which serve as precursors for steroids, ubiquinone, dolichol and isoprenoid modifications of proteins^27^,^28^. This pathway exists in most eubacteria and in plants, and is distinct from the mevalonate pathway commonly used in most eukaryotes and archaebacteria. It has thus been termed non-mevalonate pathway of isoprenoid synthesis. In this pathway, ISPD serves as a 2-C-methyl-D-erythritol-4-P cytidylyltransferase. Curiously, ISPD is the lone survivor of this pathway in mammals, since none of the other components of the non-mevalonate pathway of isoprenoid synthesis are present. *A priori*, it therefore seems very unlikely that ISPD would play a role in an alternative isoprenoid synthesis pathway.

On the other hand, some homologues of ISPD are involved in the biosynthesis of capsules in gram-negative bacteria and teichoic acids in gram-positive bacteria^29^,^30^,^31^,^32^. Here, homologues of ISPD serve as CDP-ribitol pyrophosphorylase synthesizing CDP-ribitol from CTP and D-ribitol-5-P. In many species this activity is located in the same polypeptide as a D-ribulose-5-P reductase activity, required for the synthesis of D-ribitol-5-P^30^,^31^. In turn, CDP-ribitol is used to synthesize the backbone structure of teichoic acids and cellular capsule material^33^,^34^,^35^,^36^.

The function of mammalian ISPD is unknown and ribitol phosphate structures have not been described yet in eukaryotic glycans. FKTN and FKRP seem to be localized in the Golgi apparatus, but little is known about their enzymatic activity^24^,^37^. Interestingly, FKTN and FKRP are the only mammalian proteins that carry a LicD domain, which is commonly found in bacterial enzymes that use CDP conjugates as substrates^38^,^39^. Some bacterial genomes code for fusion proteins of ISPD and proteins containing a LicD domain, suggesting that both components collaborate^18^. By analogy, we hypothesized that ISPD might generate a substrate that is subsequently used by FKTN and FKRP. In the current study, we found that ISPD serves to make CDP-ribitol and that CDP-ribitol can be used by FKTN and FKRP to transfer a ribitol phosphoryl group to α-dystroglycan. Notably, we observe that ribitol supplementation to fibroblasts from patients with ISPD mutations leads to a partial rescue of α-dystroglycan glycosylation. This indicates that ribitol supplementation should be evaluated as therapy for patients with ISPD mutations and other dystroglycanopathies.

## Results

### ISPD is a CDP-ribitol pyrophosphorylase

**Figure 1:**
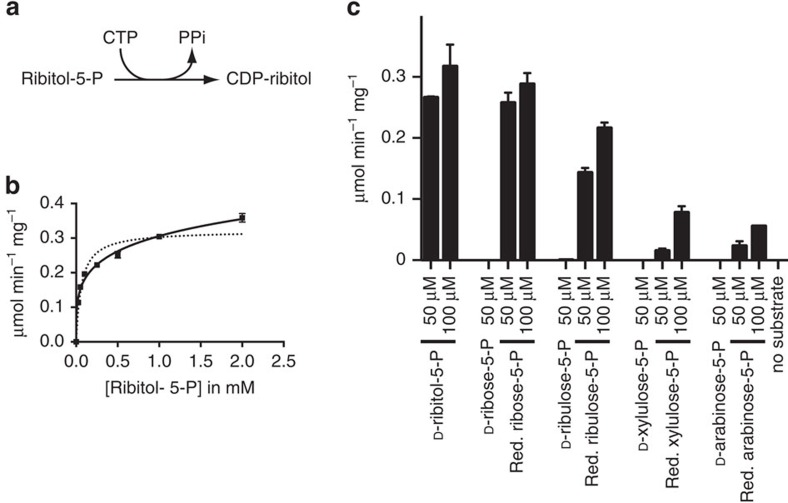
ISPD is a CDP-ribitol pyrophosphorylase. (**a**) CDP pyrophosphorylase reaction. (**b**) Activity of ISPD on CTP and D-ribitol-5-P. ISPD was incubated with the indicated concentrations of D-ribitol-5-P and 1 mM CTP for 30 min at 37 °C; CDP-ribitol was measured by HPLC. Curves were fitted to Michaelis–Menten kinetics (dotted line) and negative cooperativity (Hill coefficient=0.2; plain line). Means±s.e.m. of three independent experiments are shown. (**c**) Activity of ISPD on different pentose-phosphates and on the following pentitol-phosphates generated by borohydride reduction of pent(ul)ose-5-P: D-ribitol-5-P (reduction of D-ribose-5-P), D-arabitol-5-P (reduction of D-arabinose-5-P), D-ribitol-5-P and D-arabitol-5-P in equal amounts (reduction of D-ribulose-5-P), and equal amounts of D-xylitol-5-P and D-lyxitol-5-P (reduction of D-xylulose-5-P). Related structures are shown in Supplementary Fig. 1b. Activities were assessed in the presence of 1 mM CTP by measuring the product formation by HPLC. No more than 40% of the pent(ul)ose/pentitol-5-P was consumed at the end of the reaction. Means±s.d. (*n*=3) are shown. Three independent experiments were performed.

The N-terminal half of ISPD shows similarity to the part of *Haemophilus influenzae* Acs1 protein that is responsible for its CDP-ribitol pyrophosphorylase activity (Fig. 1a)^30^. To test whether mouse ISPD might have a similar activity, we produced a recombinant form of this protein with an N-terminal hexa-histidine tag in Sf9 cells using the baculovirus system, and purified it using metal affinity chromatography (Supplementary Material, Supplementary Fig. 1a). ISPD was incubated in the presence of UTP or CTP and different phosphorylated compounds of potential physiological relevance at 1 mM concentration. Reaction products were resolved by high-performance liquid chromatography (HPLC) using an anion exchange column and detected by their absorption at 254 and 280 nm. No activity was observed in the presence of UTP, or in the presence of CTP and 2-C-methyl-D-erythritol-4-P, which is the substrate utilized in related enzymes in the mevalonate pathway. However, we clearly observed a CDP-ribitol pyrophosphorylase activity (Fig. 1b). The substrate dependency of this reaction could best be modelled with a hyperbolic curve with a Hill coefficient of 0.2, suggesting that ISPD shows negative cooperativity with increasing D-ribitol-5-P concentrations, a characteristic that might help prevent excessive CDP-ribitol production in cells. With ≈0.5 μmol min^−1^ mg^−1^, the *V*max of ISPD is ∼30 times lower than the *V*max of bacterial enzymes that catalyse the same reaction^29^,^30^,^31^.

To evaluate whether other metabolites might be better substrates for ISPD, we tested a series of compounds including pentose phosphates or pentitol phosphates (see Supplementary Table 1 for a list of compounds that did not show any activity). At 1 mM concentration, ISPD's activity on D-ribose-5-P, D-xylulose-5-P and D-arabinose-5-P amounted to up to 10% of the activity observed with D-ribitol-5-P. However, formation of a CDP-sugar was barely detectable above background when these sugar phosphates were tested at 50 μM (Fig. 1c). To generate pentitol phosphates, we reduced pent(ul)ose phosphates with sodium borohydride, which leads to the formation of D-ribitol-5-P (from D-ribose-5-P), D-arabitol-5-P (from D-arabinose-5-P), D-xylitol-5-P and D-lyxitol-5-P (from D-xylulose-5-P) and D-arabitol-5-P and D-ribitol-5-P (from D-ribulose-5-P; as schematized in Supplementary Fig. 1b). To account for the fact that the reduction of D-ribulose-5-P and D-xylulose-5-P leads to the production of (approximately) equal parts of two different pentitol-phosphates, we tested reduced D-ribulose-5-P and D-xylulose-5-P both at 50 and 100 μM (where each pentitol-5-P is present at ≈50 μM). The highest activity was observed with D-ribitol-5-P (either purified or obtained by reduction of D-ribose-5-P) and reduced D-ribulose-5-P (which leads to the formation of D-arabitol-5-P and D-ribitol-5-P; Fig. 1c). In contrast, activities with reduced D-xylulose-5-P (that is, a mixture of D-xylitol-5-P and D-lyxitol-5-P) and reduced D-arabinose-5-P (that is, DD-arabitol-5-P) were at least four times weaker (Fig. 1c). Collectively, these experiments support the notion that ISPD serves to make CDP-ribitol.

### Detection of a CDP-pentitol in rat muscle and mouse myotubes

**Figure 2:**
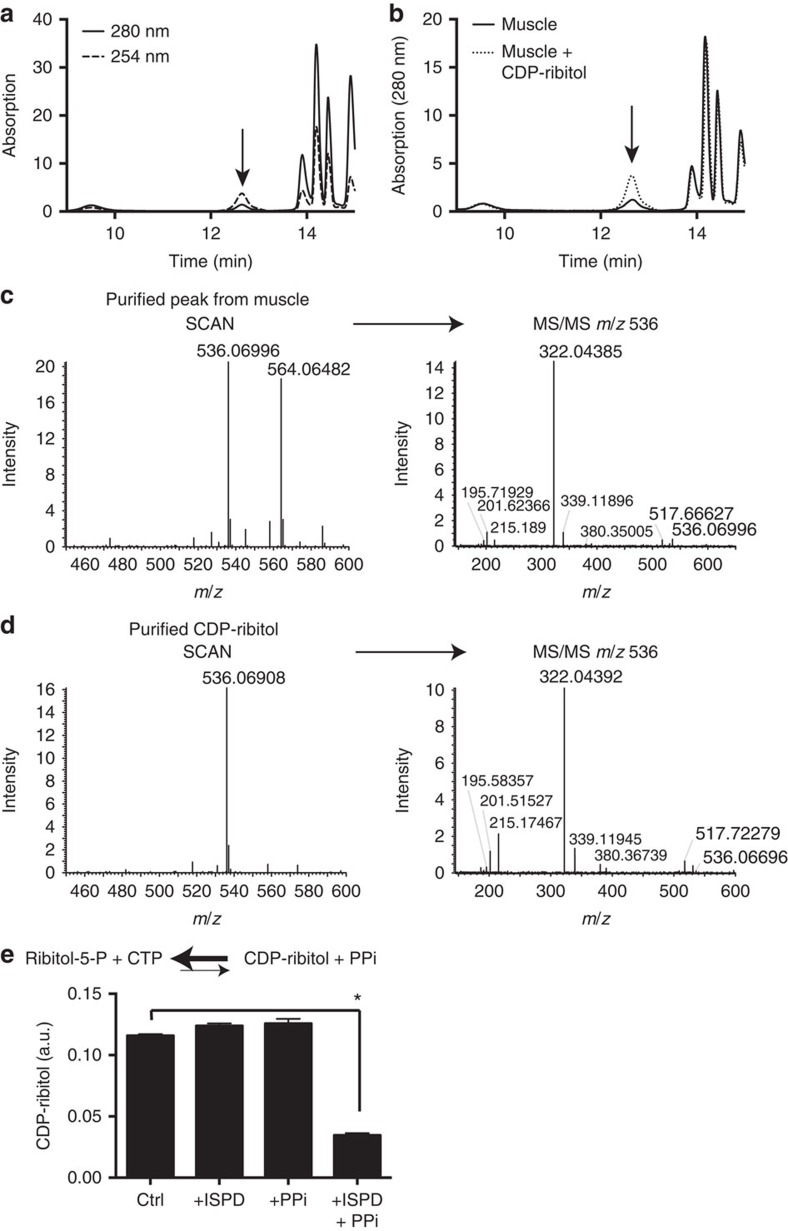
Skeletal muscle contains a CDP-pentitol, most likely CDP-ribitol. (**a**,**b**) HPLC analysis of a deproteinized rat muscle extract on a strong anion exchanger monitored by measuring absorption at 280 and 254 nm. CDP-ribitol was spiked in the run shown in (**b**). (**c**,**d**) Mass-spectrometric analysis of the putative CDP-derivative purified from (**c**) rat muscle, and of (**d**) synthetic CDP-ribitol (left panels, ‘SCAN'), as well as fragmentation pattern of the ions with *m/z* 536 (right panels, ‘MS/MS'). The ion with *m/z* 564.06482 in the sample derived from muscle most likely corresponds to CDP-glucose (Supplementary Fig. 2). (**e**) Partial conversion of the CDP-sugar peak purified from muscle by incubation with purified human ISPD in the presence of inorganic pyrophosphate (PPi). Peak areas were normalized to the total area of all peaks at 280 nm (a.u., arbitrary units). Means±s.d. of three separate reactions are shown. Three independent experiments were performed. Asterisk indicates p<0.05 in Student's *t*-test.

We, therefore, wondered whether CDP-ribitol is actually present in cells. To answer this question, we analysed samples from rat muscle (Fig. 2a). Samples were deproteinized and nucleotides were analysed by HPLC. Interestingly, we observed a peak of unknown identity that showed a ratio of the absorptions at 254 and 280 nm suggestive of a cytidine nucleotide (Fig. 2a). Spiking in of CDP-ribitol demonstrated that this peak indeed eluted at the retention time expected for CDP-ribitol (Fig. 2b).

To further characterize this peak, we purified this metabolite from rat muscle successively on a strong anion exchange column (Q-Sepharose), a gel filtration matrix (Biogel P2), and a porous graphite column (Hypercarb column). Samples were then analysed by direct injection into a high mass-accuracy mass spectrometer. Two major parent ions were detected. The first ion showed an exact mass as well as a fragmentation pattern consistent with a CDP-pentitol (Fig. 2c) and identical to those obtained for CDP-ribitol (Fig. 2d). The second ion was identified as a CDP-hexose and likely represents CDP-glucose, which coelutes with CDP-ribitol and is known to result from the side activity of UDP-glucose pyrophosphorylase on CTP and glucose-1-P (Supplementary Fig. 2)^40^. Collectively, these data provide strong evidence that muscle cells contain a CDP-pentitol. In contrast, we did not observe any ion with the predicted mass for CDP-2-C-methylerythritol, further supporting the notion that mammalian ISPD does not catalyse the reaction that related proteins perform in non-mevalonate isoprenoid metabolism^28^.

To address the question whether the CDP-pentitol in muscle might be produced by ISPD, we took advantage of the reversibility of the CDP-ribitol pyrophosphorylase reaction (Fig. 2e). To drive the reverse reaction, we incubated muscle extracts in the presence or the absence of inorganic pyrophosphate and/or ISPD. The resulting products were analysed again by HPLC. As shown in Fig. 2e, ISPD was indeed capable of reducing the level of the CDP-pentitol by up to 70%. Given the fact that we had found this peak to be composed of both CDP-ribitol and CDP-glucose (Fig. 2c), we conclude that ISPD is likely responsible for the formation of the CDP-pentitol part of this peak.

### Production of CDP-ribitol is the cellular function of ISPD

**Figure 3:**
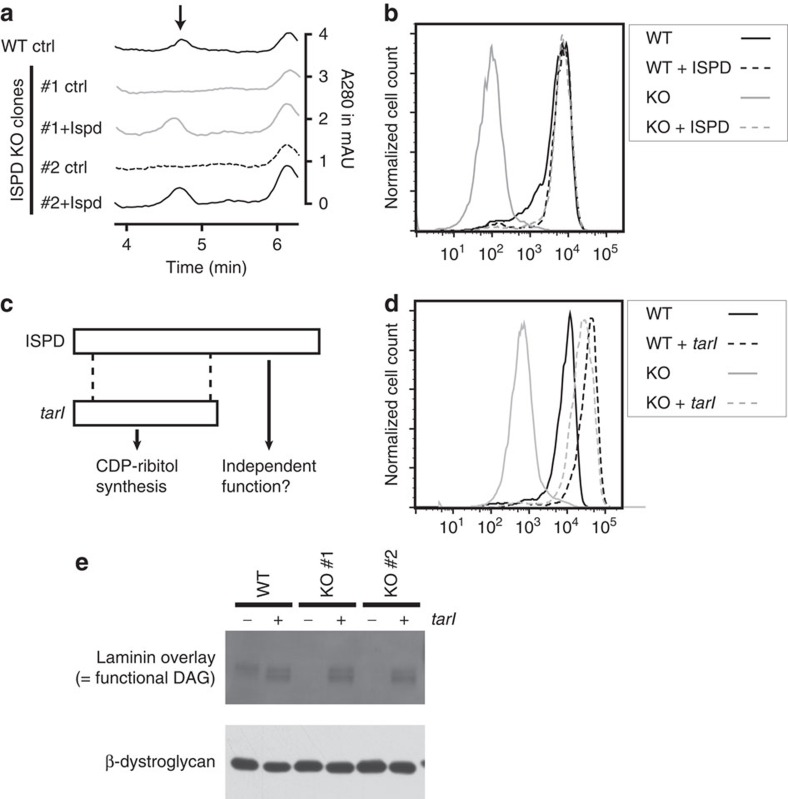
Reduced CDP-pentitol levels and α-dystroglycan glycosylation in ISPD KO cells can be corrected by a bacterial CDP-ribitol pyrophosphorylase. (**a**) HPLC analysis of WT HAP1 cells and of two ISPD KO clones that have been complemented or not with mouse ISPD. The arrow indicates the expected retention time for CDP-ribitol. (**b**–**d**) Assessment of α-dystroglycan glycosylation by flow cytometry using antibody IIH6 in wild-type cells and one ISPD KO clone, in which mouse ISPD (**b**) or *Streptococcus pneumonia* tarI (**d**) was expressed. The N-terminal part of ISPD shares similarity with *S. pneumoniae* tarI, a CDP-ribitol pyrophosphorylase (**c**). Additional ISPD KO clones are shown in Supplementary Fig. 4. (**e**) Laminin overlay analysis was performed in wild-type cells and two ISPD knockout clones completemented or not with *S. pneumoniae* tarI. Western blot analysis for β-dystroglycan is shown as loading control.

To demonstrate unequivocally that ISPD is responsible for the production of this peak, we generated knockout cell lines for ISPD in the cell line HAP1 using the CRISPR/Cas9 double-nickase system (Supplementary Fig. 3)^41^. Inactivation of ISPD led to the disappearance of the peak corresponding to a CDP-pentitol in HPLC, and re-expression of ISPD led to a re-appearance of this peak (Fig. 3a). Furthermore, inactivation of ISPD led to a reduction of fully glycosylated α-dystroglycan, which can be measured with the glyco-epitope-directed antibody IIH6 (ref. 42; Supplementary Fig. 4). In turn, re-expression of ISPD brought glycosylation back to normal levels (Fig. 3b).

The N-terminal part of ISPD shows homology to bacterial CDP-ribitol pyrophosphorylases (Fig. 3c). Curiously, disease-associated mutations in *ISPD* are not strictly limited to the N-terminal part (refs 18, 20, personal data N.S.). It was, therefore, possible that the C-terminal region had additional catalytic activity that might somehow be required for the glycosylation of α-dystroglycan. We reasoned that if a catalytic activity (distinct from the CDP-ribitol pyrophosphorylase activity) was performed by the C terminus of ISPD, glycosylation of α-dystroglycan in ISPD mutant cells should not be restored by the expression of bacterial homologues of ISPD (which synthesize CDP-ribitol required for capsule formation). To test this, we introduced a codon usage-adjusted version of TarI, a *bona fide* CDP-ribitol pyrophosphorylase from *Streptococcus pneumoniae*, into ISPD mutant cells^29^. As shown in Fig. 3d, expression of TarI completely rescued α-dystroglycan glycosylation as assessed by flow cytometry using the IIH6 antibody. Furthermore, laminin-overlay assays demonstrate that this α-dystroglycan is competent in ligand binding (Fig. 3e), consistent with the notion that the CDP-ribitol pyrophosphorylase activity of ISPD is required for correct α-dystroglycan glycosylation.

Overall, we conclude that muscle cells contain a CDP-pentitol that is synthesized by ISPD. Given that we had identified D-ribitol-5-P as best substrate for ISPD and that a bacterial CDP-ribitol pyrophosphorylase can substitute for ISPD, we conclude that ISPD's main function is to synthesize CDP-ribitol, which in turn is required for α-dystroglycan glycosylation.

### CDP-ribitol synthesis from ribose via an unknown reductase

**Figure 4:**
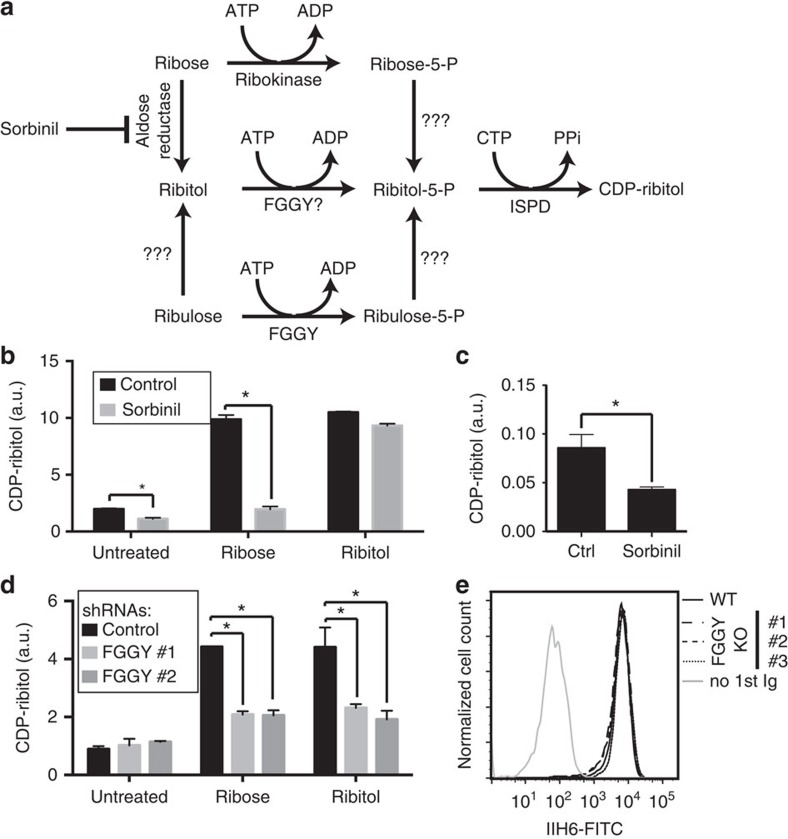
Involvement of a Sorbinil-sensitive aldose reductase and the kinase FGGY in the formation of CDP-ribitol. (**a**) Potential pathways of D-ribitol-5-P synthesis from common cellular metabolites. (**b**) CDP-ribitol levels in ISPD-overexpressing HEK293 cells incubated without or with 3 mM D-ribose or ribitol, without or with 100 μM Sorbinil, an aldose reductase inhibitor. (**c**) CDP-ribitol levels in the skeletal muscle of mice treated (*n*=3) or not (*n*=4) with Sorbinil for 6 days (means±s.e.m., asterisk indicating *P*<0.05 in Student's *t*-test). Note that the ‘CDP-ribitol' peak also comprises some CDP-glucose, as shown in the MS analysis in Fig. 2. (**d**) Effect of ribose and ribitol on the ‘CDP-ribitol' level in ISPD-overexpressing HEK293 cells incubated in the presence of 2 different shRNAs targeting FGGY or a control shRNA. (**e**) α-dystroglycan glycosylation in three different HAP1 cell line clones carrying CRISPR-Cas9 double-nickase-induced mutations in FGGY, as determined by flow cytometry using the IIH6 antibody. **b**,**d** show means±s.d. (*n*=3), and asterisks indicate *P*<0.05 in Student's *t*-test. In all panels, the area of the CDP-ribitol peak was normalized to the total area of all peaks observed at 280 nm (a.u., arbitrary units).

While our data provide evidence that ISPD serves to produce CDP-ribitol from CTP and D-ribitol-5-P, the latter is not known to be present in mammalian cells. Several potential pathways for the biosynthesis of D-ribitol-5-P are conceivable (Fig. 4a). Bacterial homologues of ISPD sometimes exist as fusion proteins with reductases that convert D-ribulose-5-P to D-ribitol-5-P^30^. To address the question whether the production in mammalian cells follows a similar path, we treated mammalian cell lines with the aldose reductase inhibitor Sorbinil and measured nucleotides by HPLC. In these experiments, cell lines overexpressing ISPD were used to facilitate detection of CDP-ribitol at baseline. We observed that Sorbinil reduced baseline CDP-ribitol levels, as well as the increase of CDP-ribitol induced by incubation in the presence of D-ribose (Fig. 4b). Likewise, we observed reduced CDP-ribitol levels in mouse muscle on treatment with Sorbinil (Fig. 4c). This suggested that reduction of D-ribose or one of its downstream metabolites might be an essential step in the formation of CDP-ribitol. At present, it is not clear whether this reduction takes place on D-ribose-5-P, D-ribulose-5-P or D-ribose, and the molecular identity of this reductase is still unknown.

Interestingly, treatment of cells with ribitol led to an increase of CDP-ribitol levels irrespective of the presence or the absence of Sorbinil (Fig. 4b, last two bars). This raised the possibility that in this particular cell line, ribitol might be directly phosphorylated by a kinase. On the basis of its similarity with prokaryotic ribulokinases, we focused on the kinase FGGY^43^. Indeed, recombinant FGGY showed not only strong ribulokinase activity but also weak ribitol kinase activity (unpublished data). To evaluate whether FGGY contributes to CDP-ribitol synthesis, we knocked-down FGGY using two different short hairpin RNAs (shRNAs) in 293 human embryonic kidney (HEK293) cells and found that this almost completely prevented the increase in CDP-ribitol levels observed on treatment of cells with ribitol (Fig. 4d). In contrast, knockdown of FGGY did not have any effect on CDP-ribitol levels in the absence of ribitol supplementation. This suggested that either our knockdown efficiency was not sufficient or that the synthesis of D-ribitol-5-P through FGGY only occurs at supraphysiological concentrations obtained when we supplement ribitol to the culture medium.

To distinguish between the two possibilities, we inactivated the *FGGY* gene in HAP1 cells using the CRISPR/Cas9 double-nickase system (Supplementary Fig. 5). When analysing metabolite levels in these cell lines, we did not observe any change in CDP-ribitol levels and we did not observe any reduction in glycosylated α-dystroglycan (measured with the antibody IIH6 in flow cytometry, Fig. 4e). This indicates that at physiological concentrations of ribitol, FGGY is not required for the synthesis of CDP-ribitol in HAP1 cells. However, our data are consistent with the notion that at elevated ribitol concentrations FGGY might help increase intracellular D-ribitol-5-P concentrations (which becomes relevant below).

### FKTN and FKRP can transfer ribitol-5-P to α-dystroglycan

**Figure 5:**
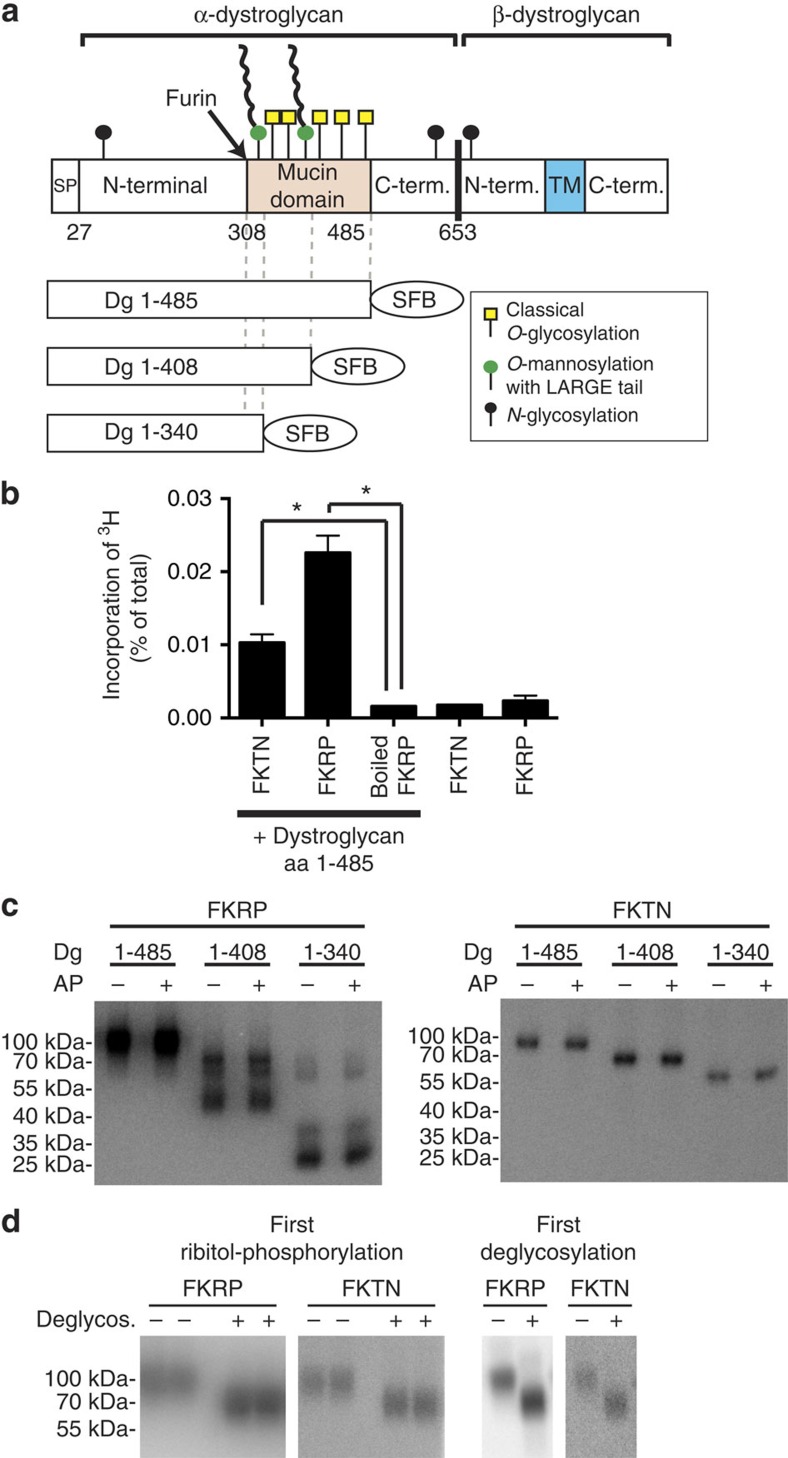
FKTN and FKRP can transfer ribitol phosphate to recombinant α-dystroglycan. (**a**) Schematic representation of α-dystroglycan fragments used in this study. (**b**) Transfer of [^3^H]-ribitol from CDP-[1-^3^H]-ribitol to α-dystroglycan (∼1 μg) by recombinant FKTN and FKRP (∼0.1 and ∼0.4 μg, respectively). After incubation for 16 h at 30 °C, proteins were recovered using streptavidin beads and incorporated radioactivity was determined with a scintillation counter. Values are means±s.d. of three biological replicates from one representative experiments out of three. Asterisks indicate *P*<0.05 in Student's *t*-test. (**c**) Incorporation of [^32^P] from [^32^P]-CDP-ribitol to different fragments of α-dystroglycan by FKTN and FKRP. After 16 h of incubation at 30 °C, proteins were incubated with (+) or without (−) alkaline phosphatase (AP) and resolved by SDS–PAGE. ^32^P was detected with a storage phosphorimager. (**d**) Effect of deglycosylation of α-dystroglycan (aa 1–485) with a ‘deglycosylation mix' on the incorporation of radioactivity from [^32^P]-CDP-ribitol catalysed by FKRP or FKTN. The deglycosylation treatment of α-dystroglycan performed either before (two left panels) or after (two right panels) ribitol-phosphorylation decreased the apparent molecular weight of α-dystroglycan, but did not reduce the incorporated radioactivity.

Bacterial homologues of FKTN and FKRP use CDP-choline or CDP-ribitol as substrates to transfer phosphorylcholine or ribitol phosphate to reaction intermediates in teichoic and capsule biosynthesis^29^,^44^. By analogy, we hypothesized that FKTN and/or FKRP might use CDP-ribitol as a substrate to transfer a ribitol phosphate group onto α-dystroglycan. To test this hypothesis, we expressed N-terminal α-dystroglycan fragments of different lengths with a C-terminal affinity tag consisting of an S-tag, two FLAG tags and a streptavidin-binding peptide in HEK293 cells (SFB-tag, (ref. 45)) (Fig. 5a). Cellular supernatants were recovered from the cultures after 5 days at confluence, and purified via Sepharose beads covalently bound to streptavidin (Supplementary Fig. 6a). In parallel, we purified FKTN and FKRP from cellular lysates via the same strategy (Supplementary Fig. 6b).

Most glycosyltransferases transfer only the sugar part from NDP-sugars onto their target glycans. In contrast, LicD domain-containing proteins also transfer the adjacent phosphate group^46^. To assess whether FKTN and/or FKRP likewise transfer a ribitol phosphate group we synthesized CDP-ribitol where either the phosphate group attached to the ribitol (^32^P), or the ribitol part itself (^3^H attached to C1) were radioactively labelled. We then incubated these reagents with purified rabbit α-dystroglycan fragments in the presence of either FKTN or FKRP, and detected radioactivity incorporated into α-dystroglycan derived from either ribitol (^3^H) or phosphate (^32^P).

In the case of ^3^H-labelled CDP-ribitol, we observed significant incorporation of ^3^H into α-dytroglycan only when FKTN or FKRP were present (Fig. 5b). In contrast, no incorporation was observed when the enzymes were heat inactivated or when α-dystroglycan was omitted (Fig. 5b). This indicated that FKTN and FKRP can indeed transfer the ribitol part of CDP-ribitol onto α-dystroglycan.

We also incubated ^32^P-labelled CDP-ribitol in similar experiments with different α-dystroglycan fragments in the presence of either FKTN or FKRP. Incorporation of ^32^P was detected by storage phosphorimaging after SDS–PAGE. As shown in Fig. 5c, both FKTN and FKRP were able to transfer ribitol phosphate from CDP-ribitol onto α-dystroglycan. When using FKRP as enzyme, we also observed [^32^P]-phosphate incorporation not only to the intact α-dystroglycan fragments but also to significantly shorter fragments of very low abundance (see the lower bands in Fig. 5c, Supplementary Fig. 7). At present, it is unclear whether this is due to a direct ribitol phosphate transfer by FKRP to these fragments, or if these bands result from breakdown of labelled fragments by a protease or glycosidase contaminating the FKRP preparation. In any case, the incorporation of ^32^P in α-dystroglycan was largely resistant to treatment with alkaline phosphatase (Fig. 5c) suggesting that the phosphate group is present as a phosphodiester protected by the ribitol group from an attack by alkaline phosphatase.

We next treated ribitol-phosphorylated α-dystroglycan fragments with a mixture of several glycosidases that remove *N*-glycans (PNGaseF from *Flavobacterium meningosepticum*^47^), and either significantly trim (neuraminidase from *Clostridium perfringens*, β-1,4-galactosidase from *Bacteroides fragilis* and β-*N*-acetylglucosaminidase from *Xanthomonas manihotis*) or remove (Endo-α-*N*-Acetylgalactosaminidase from *Enterococcus faecalis*^48^) mucin-type *O*-glycans. As shown in Fig. 5d, this treatment significantly reduced the apparent molecular weight of the proteins in SDS–PAGE, but barely reduced the ^32^P signal incorporated from [^32^P]-CDP-ribitol. This excludes *N*-glycans and the majority of *O*-glycan structures (not being *O*-mannosylated) as a point of attachment of the ribitol phosphate groups. To test whether these glycans need to be present in order for FKTN and FKRP to work, we first treated α-dystroglycan with the glycosidase mixture and then transferred ribitol phosphate groups with FKTN or FKRP. Again, under these conditions FKTN and FKRP were able to transfer ribitol phosphate (Fig. 5d). This indicates that most mucin-type *O*-glycan structures on α-dystroglycan are dispensable for the recognition by FKTN and FKRP, and do not represent the site of attachment of ribitol phosphate. Formally, we cannot make the same conclusion for *N*-glycans, since we omitted the initial denaturation step required for full activity of PNGaseF in the experiments where deglycosylation was performed before treatment with FKRP and FKTN (Fig. 5d). Given prior data that *N*-glycosylation is not involved in the assembly of the glycan deficient in dystroglycanopathies, an attachment of ribitol phosphate to *N*-glycan seems very unlikely^49^. Overall, our data are consistent with a model where FKTN and FKRP transfer a ribitol phosphate group from CDP-ribitol onto the *O*-mannosyl-glycan of α-dystroglycan.

### Incorporation of ribitol into α-dystroglycan in cells

To test whether ISPD, FKTN and FKRP collaborate in cells to transfer ribitol phosphate groups, we analysed glycans derived from recombinant α-dystroglycan by gas chromatography mass spectrometry (GC-MS) after depolymerization with methanolic HCl. Starting out with α-dystroglycan purified from HEK293 cells engineered to overexpress ISPD, we observed a peak at the retention time and with the fragmentation pattern expected for trimethylsilylated (TMS) ribitol (Supplementary Fig. 8a and b). The peak intensity increased when purified ribitol was added before derivatization, and was distinct from the peak obtained when the related pentitol arabitol was added (Supplementary Fig. 8c) indicating that the pentitol present in α-dystroglycan is ribitol. It should be noted that despite the high peak in the extracted ion chromatogram at *m/z* 307 (Supplementary Fig. 9a and b), the contribution of ribitol to the total ion current of monosaccharides derived from α-dystroglycan is small when compared with other monosaccharides (Supplementary Fig. 9d), consistent with the notion that only few ribitol phosphate groups are present in α-dystroglycan. Ribitol incorporation does not seem to be a general characteristic of glycoproteins, since no ribitol peak was observed when analysing an unrelated glycoprotein (neurofascin) that is known to contain mucin-type *O*-glycans and *O*-mannose glycans (Supplementary Fig. 9c)^50^.

**Figure 6:**
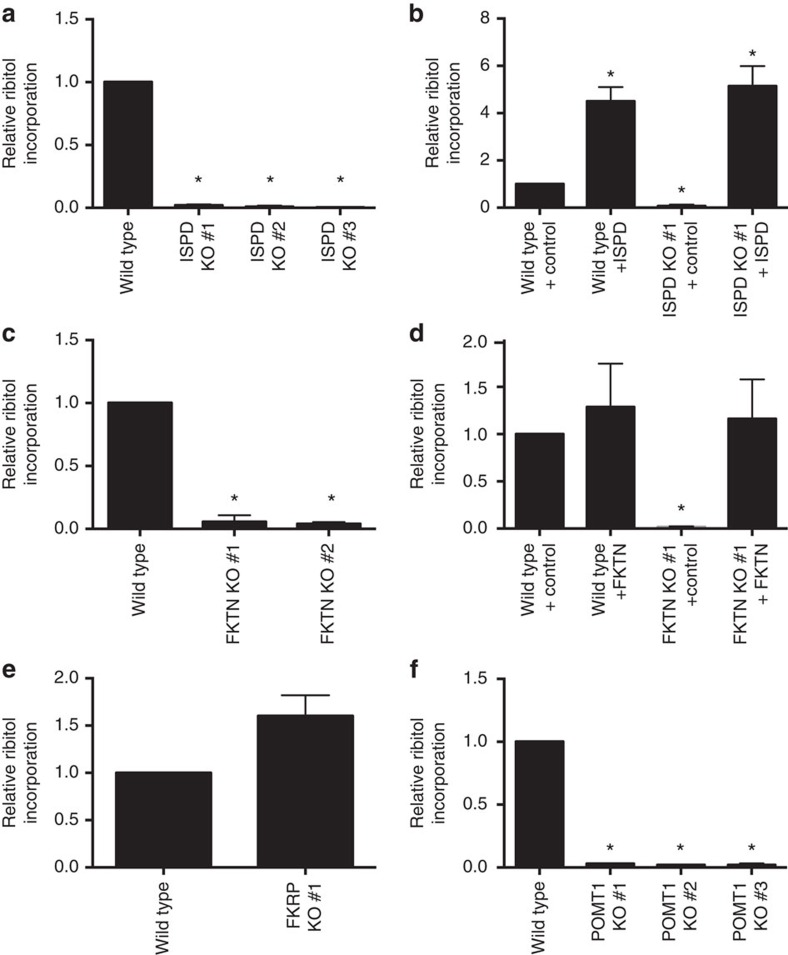
Incorporation of ribitol in α-dystroglycan in cells depends on ISPD, FKTN and POMT1. (**a**–**f**) An α-dystroglycan fragment (comprising amino acids 1–485 and a C-terminal SFB-tag) was purified by affinity chromatography from cell culture supernatants of HEK293 clones, in which the genes indicated in the figure had been inactivated. N- and O-glycans were released under non-reducing conditions, hydrolysed and derivatized with TMS before analysis by GC-MS to assess ribitol incorporation, which was normalized to methyl-glycine concentrations. Means and s.e.m. of 3–6 independent experiments are shown, and values were normalized within each experiment to levels in wild-type (control) cells. Asterisks indicate *P*<0.05 obtained from Dunnett's multiple comparisons test, compared with wild type or wild-type control cells. ISPD or FKTN knockout clones (**a**,**c**), as well as one ISPD (**b**) and one FKTN (**d**) knockout clone complemented or not with mouse Ispd (**b**) or Fktn (**d**) cDNA were analyzed. One FKRP clone (**e**) and three different POMT1 knockout clones (**f**) were analysed.

To test the role of ISPD in the incorporation of ribitol into α-dystroglycan, we knocked out *ISPD* in HEK293 cells (Supplementary Fig. 10, Fig. 6a,b). As expected, loss of ISPD led to a complete absence of ribitol in α-dystroglycan purified from these cell lines (Fig. 6a), and its presence could be restored by the re-expression of mouse Ispd (Fig. 6b). This demonstrates that ISPD plays an essential role in the introduction of ribitol into α-dystroglycan in HEK293 cells. We also note that overexpression of ISPD in wild-type cells increased ribitol incorporation into α-dystroglycan (Fig. 6b), indicating that the production of CDP-ribitol might be a limiting element of this process in our experimental system, and that many sites that could be ribitol-phosphorylated do not carry this modification in HEK293 cells.

To evaluate the role of *FKTN* and *FKRP*, we knocked out these genes in HEK293 cells (Supplementary Figs 11 and 12). Inactivation of *FKTN* led to a complete loss of ribitol incorporation (Fig. 6c), which was restored on re-expression of mouse Fktn (Fig. 6d). This indicates that FKTN is essential for the incorporation of ribitol into α-dystroglycan in HEK293 cells (likely via a transfer of a ribitol phosphate group). In contrast, ribitol incorporation into α-dystroglycan purified from an FKRP knockout clone was maintained (Fig. 6e). At first sight this could be interpreted as evidence that FKRP does not play a role in transferring ribitol phosphate in HEK293 cells at all. It could also lead to the hypothesis that the ribitol phosphate transfer mediated by purified FKRP *in vitro* (Fig. 5b,c) could be a consequence of a contamination of this preparation with FKTN. As shown in Supplementary Fig. 6c, FKTN was not detectable in purified FKRP by western blotting, confirming that FKRP can perform a ribitol phosphate transfer reaction. Given that CDP-ribitol levels seem to be limiting ribitol incorporation into a-dystroglycan (see above, Fig. 6b), FKRP and FKTN likely compete for this substrate, and loss of FKRP might lead to an increase in ribitol incorporation by FKTN. The recombinant α-dystroglycan used in *in vitro* assays has been produced from cells that express FKTN, and is therefore partially modified by FKTN. Given the action of FKRP on these substrates *in vitro*, it is tempting to speculate that FKRP can only transfer a ribitol phosphate group once FKTN has performed its action.

In this context, we need to note that in the presented experiments we only analysed ribitol, not phosphate, incorporation into α-dystroglycan. Given that FKTN and FKRP transfer a ribitol phosphate group *in vitro*, we conclude that ISPD, FKTN and FKRP likely collaborate to transfer a ribitol phosphate group onto α-dystroglycan in cells. At present, we do not know where this group is added, but we do notice that ribitol incorporation in *POMT1* knockout cells is abolished, indicating that *O*-mannosylation is required for the attachment of ribitol phosphate (Fig. 6f, Supplementary Fig. 13).

### Ribitol partially rescues glycosylation in ISPD mutant cells

Mutations in ISPD associated with dystroglycanopathies are not all predicted to completely inactivate ISPD's catalytic function^18^,^20^. Hence, we wondered whether increasing the cellular concentration of its substrate, D-ribitol-5-P, could rescue part of α-dystroglycan glycosylation.

**Figure 7:**
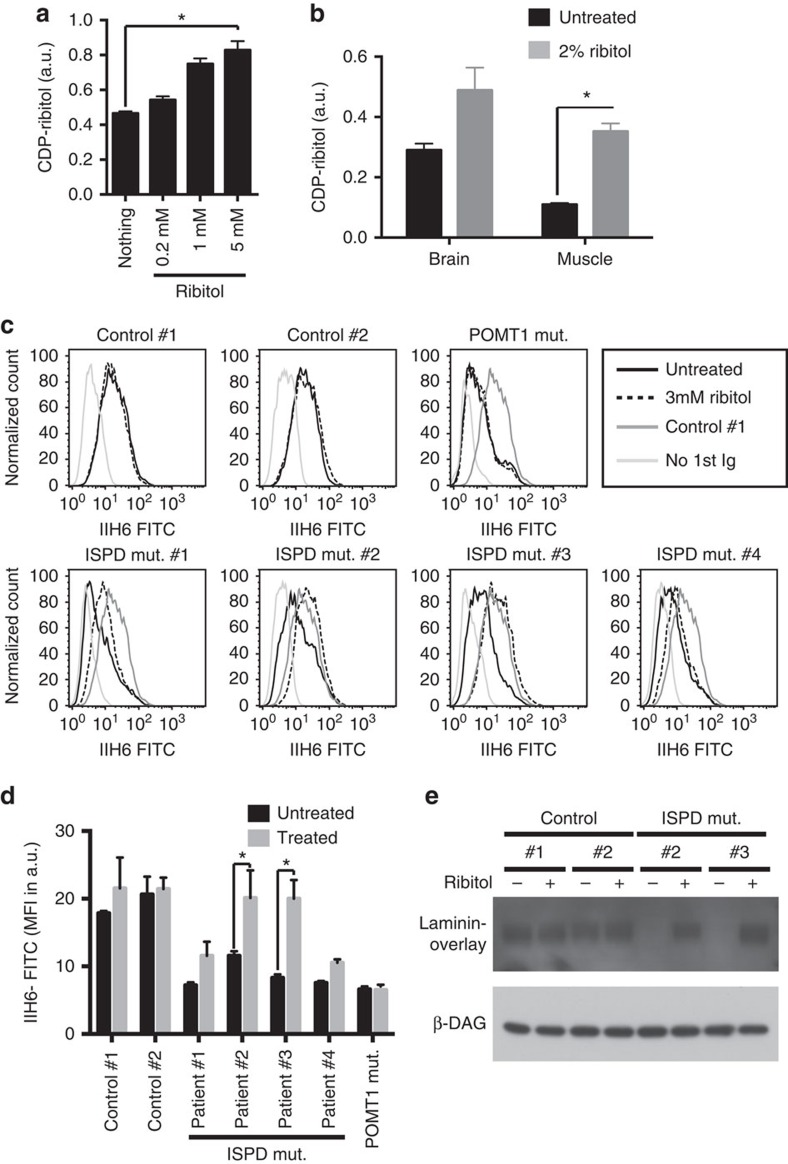
Effect of ribitol supplementation on the CDP-ribitol levels and α-dystroglycan glycosylation. (**a**) Differentiated C2C12 myotubes were incubated overnight with the indicated concentrations of ribitol and CDP-ribitol levels were determined by HPLC. Means±s.d. (*n*=3) are shown and asterisk indicates *P*<0.05 in Student's *t*-test. (**b**) Mice were given water containing 2% ribitol for three weeks. CDP-pentitol levels in organs were assessed by HPLC. Means±s.e.m. are shown (*n*=4 untreated, *n*=3 treated) and asterisk indicates *P*<0.05 in Student's *t*-test. (**c**,**d**) Fibroblasts from patients with mutations in the indicated genes were treated for 96 h with 3 mM ribitol. α-Dystroglycan glycosylation was assessed by flow cytometry using the IIH6 antibody. Histograms (**c**) and mean fluorescence intensity (MFI; means±s.d. for *n*=3 samples) (**d**) are shown for one representative experiments out of 3. Asterisks indicate *P*<0.05 obtained from Dunnett's multiple comparisons test. A negative control run without first antibody is shown in light grey in all panels of **c**. The signal observed with IIH6 antibody in control #1 is included as a reference in all panels corresponding to mutated fibroblasts. (**e**) Laminin overlay was performed on dermal fibroblasts obtained from control patients as well as ISPD mutant patients #2 and #3 on treatment with 3 mM ribitol for 96 h. Western blot analysis for β-dystroglycan is shown to demonstrate equal loading.

We first addressed the question whether supplementation of ribitol would lead to an increase of CDP-ribitol levels in cells, and treated differentiated C2C12 myotubes with ribitol. Under these conditions, we observed a dose-dependent increase in CDP-ribitol (Fig. 7a). This observation indicated that D-ribitol-5-P concentrations in cells do not saturate ISPD activity. In other words, increases in cellular D-ribitol-5-P concentrations are expected to lead to increased CDP-ribitol concentrations.

To address the same question *in vivo*, we supplemented the drinking water of mice for 3 weeks with 2% ribitol. As shown in Fig. 7b, this led to a more than threefold increase in CDP-ribitol levels in muscle, suggesting that increased extracellular ribitol levels *in vivo* could also lead to an increase in cellular CDP-ribitol levels.

To test whether supplementation with ribitol could improve deficient α-dystroglycan glycosylation in patients, we incubated fibroblasts derived from patients with ISPD mutations (Supplementary Table 2) in the presence or in the absence of 3 mM ribitol for 4 days. As shown in Fig. 7c and quantified in Fig. 7d, supplementation of ISPD mutant fibroblasts with ribitol significantly increased α-dystroglycan glycosylation in fibroblasts from two patients out of four tested, as assessed by flow cytometry using the IIH6 antibody, reaching almost the levels observed in fibroblasts obtained from control patients. In contrast, ribitol supplementation induced no difference in fibroblasts from healthy controls or from patients carrying mutations in the *POMT1* gene, which codes for the enzyme catalysing the initial step of *O*-mannosylated glycan synthesis^51^. To confirm that α-dystroglycan in *ISPD* mutant fibroblasts is functional, we performed a laminin overlay assay in the two responsive *ISPD* mutant fibroblast lines and in two lines from normal controls. As shown in Fig. 7e, laminin binding in ISPD mutant cell lines was completely absent and was largely restored on treatment with ribitol.

Overall, our data suggest that dietary supplementation with ribitol should be evaluated as a therapeutic option for patients with *ISPD* mutations. Furthermore, an increase in CDP-ribitol levels might also prove beneficial in patients carrying mutations in the *FKTN* and *FKRP* genes.

## Discussion

Homologues of ISPD have been found to be involved in two main reactions in nature: the formation of CDP-2-C-methylerythritol or the formation of CDP-ribitol^28^,^30^. We did not observe any activity of mouse Ispd with 2-C-Methyl-D-erythritol-4-P, the substrate used by ISPD homologues in the non-mevalonate pathway of isoprenoid synthesis^28^. The conclusion that ISPD is not involved in this pathway is also supported by the absence of all its other enzymes in mammalian genomes. In contrast, we did observe considerable activity of ISPD with D-ribitol-5-P, which led to the formation of CDP-ribitol, and much less activity if any with other pentitol phosphates or with hexose, pentose and hexitol phosphates. Consistent with D-ribitol-5-P being the physiological substrate of ISPD, we observed a peak that coeluted with CDP-ribitol when skeletal muscle extracts were analysed by HPLC. Mass spectrometry showed an exact mass and a fragmentation pattern identical to those of CDP-ribitol. Furthermore, the peak isolated from muscle could be degraded by the backward reaction catalysed by recombinant mouse Ispd in the presence of PPi and it was absent in ISPD knockout cells, supporting the conclusion that it consists of a CDP-pentitol produced by ISPD.

Unfortunately, most CDP-pentitols would likely yield a fragmentation pattern in mass spectrometry that closely resembles the one obtained for CDP-ribitol. This means that still another pentitol phosphate might be a better substrate for Ispd. This being said, we did test those that can be made by a single reduction reaction from classical metabolic intermediates, and D-ribitol-5-P clearly emerged as the best substrate. Given that our CDP-ribitol standard was synthesized using ISPD, it is also conceivable that the C-terminal part of unknown function might lead to an unknown isomerization reaction. However, this hypothesis is very unlikely given that a GC-MS analysis of the hydrolysis products of α-dystroglycan purified from HEK293 cells revealed the presence of ribitol (which can be separated from the closely related pentitols arabitol and xylitol using this technique). Furthermore, reintroduction of the *bona fide* CDP-ribitol pyrophosphorylase TarI into ISPD knockout cells restored functional α-dystroglycan glycosylation (as measured by laminin overlay; Fig. 3). The most parsimonious explanation for all these results is therefore that D-ribitol-5-P is the physiological substrate for ISPD.

At present, the biochemical pathway leading to the production of ribitol-5-P still needs to be characterized, although our data indicate that some redundancy might exist in the synthesis of this metabolite (Fig. 4). We observe that a Sorbinil-sensitive reductase is likely involved for the formation of ribitol-5-P from intracellular metabolites, but that formation of ribitol-5-P from exogenously added ribitol is catalysed by the FGGY protein.

Synthesis of CDP-ribitol in bacteria is required for structural components of teichoic acids and capsules, which represent a significant fraction of the bacterial cell mass^33^,^34^,^35^. In contrast, so far α-dystroglycan is the only eukaryotic protein where ribitol phosphorylation has been found. On the basis of the total phosphate content of this protein, only a very small number of ribitol phosphate residues (that is, between 4 and 7) can be present per α-dystroglycan molecule^13^,^14^. Furthermore, even if some other proteins (for example, among the recently identified *O*-mannosylated proteins^52^,^53^) would be found to be modified by ribitol phosphorylation, they would likely not represent a large fraction of cellular mass. Hence, it is not surprising that the *V*max of murine Ispd is ∼30 times lower than the one observed with bacterial CDP-ribitol pyrophosphorylases^29^,^30^.

Ribitol phosphate groups in bacterial teichoic acids and capsules often carry secondary modifications^33^,^34^,^35^,^36^. By analogy, it is tempting to speculate that this structure in α-dystroglycan would represent a scaffold for the attachment of the xylose- and glucuronic acid-containing polymer by LARGE and B4GAT1 (refs 15, 16, 17). In contrast to the cyclic, pyranose forms of sugars commonly observed in mammalian glycoproteins, the linear structure of ribitol is much more flexible. This flexibility could represent an advantage for the assembly of the α-dystroglycan glycan by allowing it to mediate non-directional interactions with extracellular matrix proteins such as laminin.

Mature α-dystroglycan contains between four and seven phosphate groups^13^,^14^. Part of this phosphate is esterified to C6 of a mannose residue^14^, as a result of the action of POMK/SGK196 in the endoplasmic reticulum^11^,^54^ and some is present in phosphorylated N,N'-diacetyllactosamine groups^55^. Interestingly, part of the phosphate contained in mature α-dystroglycan is protected from access by phosphatases by an unknown modification^14^. The nature of this modification is unclear at present, but it is tempting to speculate that the inaccessible phosphate groups result from the reactions catalysed by FKTN and FKRP. Like related enzymes of the LicD family that incorporate polyol phosphates in teichoic acid^46^ and mannosylphosphate in yeast mannans^39^, FKTN and FKRP are indeed expected to transfer a ribitol phosphate group to an acceptor in such a way that a phosphodiester bond is formed. Both FKRP and FKTN can transfer ribitol phosphate groups from CDP-ribitol to α-dystroglycan (Figs 5 and 6) *in vitro*, but these activities are not redundant *in vivo*, since patients with mutations in either FKRP or FKTN show a deficiency in mature α-dystroglycan glycosylation. On the basis of our results, FKTN can transfer ribitol phosphate onto α-dystroglycan independently of FKRP, since ribitol incorporation into α-dystroglycan is maintained in an FKRP knockout cell line (Fig. 6d). In turn, we demonstrate that the activity of FKRP *in vitro* is not caused by a contamination by FKTN (Supplementary Fig. 6c), indicating that FKRP can transfer ribitol phosphate independently of FKTN. Future studies will have to clarify the precise sequence of action of FKTN and FKRP and the precise sites where the ribitol phosphate groups are attached in the glycan of α-dystroglycan and potentially of other glycoproteins. Eventually, these studies will help us understand the structure of the α-dystroglycan glycan and will shape our understanding of the function of ribitol phosphorylation in vertebrate physiology.

Mutations in ISPD, FKTN and FKRP lead to dystroglycanopathies with severe neuromuscular symptoms. We demonstrate that ribitol supplementation in a subset of fibroblasts carrying ISPD mutations partially rescues α-dystroglycan glycosylation. Futhermore, we show that dietary supplementation to wild-type mice increases CDP-ribitol levels in mouse muscle. Previously, alimentary supplementation with mannose or galactose has been successful in treating patients with phosphomannose isomerase or phosphoglucomutase 1 deficiencies, respectively^56^,^57^. Our data indicate that alimentary ribitol supplementation should be evaluated as a novel therapeutic approach for patients carrying ISPD mutations.

After this work had been first submitted for publication, two articles appeared on the same subject^58^,^59^. Both of them independently showed that ISPD is a CDP-ribitol pyrophosphorylase. In addition, Kanagawa^58^ showed that FKTN and FKRP are ribitol-5-P transferases that modify the phosphorylated CoreM3 glycan of α-dystroglycan. These findings are in excellent agreement with ours. Kanagawa *et al*.^58^ further showed that the site of attachment of the first ribitol-5-P unit is the third carbon of the GalNAc residue of the CoreM3 trisaccharide. These findings agree with our observation that POMT1 is indispensable for the attachment of ribitol-phosphates by FKTN and FKRP to α-dystroglycan.

## Methods

### Cloning and plasmid construction

We amplified the Ispd open reading frame from mouse brain complementary DNA (cDNA) using the primers 5′-GCGCTCTGCCTGCTATGGAGCCTG-3′ and 5′-GTCTGTGTTCTTCATGCCACCAGG-3′. The resulting PCR product was cloned into the vector pJET 1.2-blunt (Fermentas) giving rise to plasmid pIG223. Using this vector as a template, a PCR product with the sense primer 5′-TTATTACATATGGAGCCTGGGCCGTGCAGCA-3′ and pJET 1.2 reverse primer (Fermentas) was generated, digested with the restriction endonucleases *Nde*I and *Bgl*II, and inserted into the *Nde*I–*Bam*HI sites of the bacterial expression vector pET28a resulting in the vector pOH171. To allow expression in the baculovirus system, the open reading frame was shuttled into the *Sma*I–*Xho*I sites of the vector pOET3 (Oxford expression technologies) resulting in the vector pIG252.

To generate doxycycline-inducible mouse Ispd expression constructs, we used a lentiviral vector based on the plasmid pTRIPZ empty (Openbiosystems), in which we inserted a PCR product containing the mouse Ispd ORF (primers 5′-ATACATACCGGTACCATGGAGCCTGGGCCGTGCAGCA-3′ and 5′-TTATTACTCGAGTCATGCCACCAGGAGCTGCCCAACA-3′) between the *Xho*I and *Age*I sites, leading to plasmid pIG249. For constitutive expression of mouse Ispd selectable with the antibiotic hygromycin, we transfered the open reading frame between the *Nhe*I and *Bsr*GI sites of a bicistronic lentiviral vector (pOH425) derived from the plasmid pGIPZ empty (Openbiosystems); this gave rise to plasmid pOH460.

Mouse Fktn and Fkrp were amplified with flanking *Nhe*I and *Bsr*GI sites by PCR from brown adipose tissue and thymus cDNA, respectively, using the primers NheI_mFKTNs 5′-ATACATGCTAGCCACCATGAGTAGAATCAATAAGAACGTG-3′, *Bsr*gI_mFKTNas 5′-TTATATTGTACAGTACAACTGGATAACCTCATCC-3′, as well as *Nhe*I_mFKRPs 5′-ATACATGCTAGCCACCATGCGGCTCACCCGCTGCTG-3′ and *Bsr*gI_mFKRPas 5′-TTATATTGTACAACCGCCTGTCAAGCTTAAGAGTGC-3′. The amplification products were inserted in the lentiviral vector pOH233-1 (containing a C-terminal SFB-tag consisting of an S-tag, two FLAG tags and a SBP)^60^ opened with *Nhe*I-*Bsr*GI. This gave rise to pOH381 and pOH382, respectively. In parallel, a lentiviral expression vector for mouse Fktn was generated by inserting a PCR product obtained with primers mFktn_s 5′- ATACATGCTAGCCACCATGAGTAGAATCAATAAGAACGTG-3′ and mFktn_as 5′-AAAATGTACAGGTCAGTACAACTGGATAACCTCATCC-3′, and pOH381 as template, between the *Nhe*I and *Bsr*GI sites of the plasmid pGTB5001 (a lentiviral vector driving expression of the gene of interest and a G418 resistance gene from a bicistronic transcript) leading to pUB44.

Fragments of rabbit α-dystroglycan cDNA were amplified by PCR from the construct DgFc5 (a generous gift from Stefan Kunz^61^) using primer pair T7_sense_*Xba*I 5′-ATACATTCTAGAATACGACTCACTATAGGGA-3′ and rev_DG1_340_*Bsr*GI 5′-ATACATTGTACAAGGCACGATCCTGGACGG-3′, rev_DG1_408_*Bsr*GI 5′-ATACATTGTACAAGGAATGGTCACCGTTGCACGAAT-3′ and rev_DG1_485_*Bsr*GI 5′-ATACATTGTACAGCTGGTGGTGGTGCGGATACGAGT-3′, corresponding to amino acids 1–340, 1–408 and 1–485 of α-dystroglycan. The PCR products were subcloned as described above into the pOH233 vector, resulting in plasmids pOH347, pOH348 and pOH349, respectively.

The cDNA sequence of *Streptococcus pneumoniae* tarI was optimized for mammalian codon-usage and PCR-amplified from a synthetic geneblock (IDT) using primers tarI_sense 5′-ATACATAGCTAGCCACCATGATCTACGCC-3′ and tarI_reverse 5′-TATAAGGTACCTCAGTCCTTCTCGAT-3′. The product was digested with *Nhe*I and *Acc*65 and ligated into between *Nhe*I and *Bsr*GI sites of the plasmid pGTB5002 (a lentiviral vector driving expression of the gene of interest and a puromycin resistance gene from a bicistronic transcript) yielding pIG292.

Guide RNA targeting human *ISPD*, *FGGY*, *FKTN*, *FKRP* and *POMT1* genomic sequences (targeted sites ISPD #1 CCCACCCCGAAGCAATTCTGCCC, ISPD #2 GCTCATCAGCTACACCCTACAGG, FGGY #1 CCAGTTCAACCACCATGAGCAGT, FGGY #2 CTGTGTTGTCACAAAGGTATGGG, FKTN #1 CCAAGTGAGCAGCACAGACTAAT, FKTN #2 GAATCAATAAGAACGTGGTTTTGG, FKRP #1 CCTAGTACCTGATGGGGCGCGGGC, FKRP #2 TGGCCTGCTGGAGCGCATGGTGG, POMT1 #1 CCAGAATACAGTAGCAACGTGCC and POMT1 #2 GCCTGCTGCCAGCACTCGCGGGG) were introduced into the *Bbs*I site of the vector pX462 as described before^41^.

Vectors driving doxycycline-inducible expression of two different shRNAs targeting human FGGY (FGGY1 and FGGY2) were generated by inserting PCR-amplified oligonucleotide pairs into the vector pTRIPZ empty (Openbiosystems)^62^. Briefly, pairs of oligonucleotides (hFGGY1_s 5′-TGCTGTTGACAGTGAGCGACATCGAGCAGTCAGTCAAGTTTAGTGAAGCCACAGATGTA-3′ and hFGGY1_as 5′-TCCGAGGCAGTAGGCACCATCGAGCAGTCAGTCAAGTTTACATCTGTGGCTTCACTA-3′, hFGGY2_s 5′-TGCTGTTGACAGTGAGCGCGCAGATAATTACAGCAAAATATAGTGAAGCCACAGATGTA-3′ and hFGGY2_as 5′-TCCGAGGCAGTAGGCATGCAGATAATTACAGCAAAATATACATCTGTGGCTTCACTA-3′) were annealed and extended in the presence of PfuI polymerase (Thermofisher) using the PCR program 5 min at 95 °C, followed by 10 cycles with the following steps: 30 s at 95 °C, 30 s at 60 °C and 30 s at 72 °C. The resulting fragments were then PCR-amplified with primers miR30PCRXhoIF_s 5′-CAGAAGGCTCGAGAAGGTATATTGCTGTTGACAGTGAGCG-3′ and miR30PCREcoRIF_rev 5′-CTAAAGTAGCCCCTTGAATTCCGAGGCAGTAGGCA-3′, digested with *Xho*I and *Eco*RI, and ligated into a pTRIPZ empty plasmid (GE healthcare), yielding pIG271 and pIG273, respectively^62^.

### Production of radioactive compounds

*[^32^P]-CDP-ribitol synthesis*. 5 mM D-ribose was incubated with 50 nM [^32^P]-γATP in 25 mM Hepes, pH 7.4, 1 mM MgCl_2_, 25 mM KCl and 25 μM ATP-Mg^2+^ in the presence of 10 units of ribokinase^63^ for 60 min at 30 °C. After addition of NaBH_4_ to a final concentration of 0.333 M, the reaction mixture was incubated at room temperature (RT) for 2 h, titrated with HCl to reach a pH of 5.0, diluted fourfold and loaded on a 1 ml DOWEX AG-1X8 (200–400 Cl^−^ form) column. Elution was performed with increasing concentrations of KCl and [^32^P]-ribitol-5-P eluted at a concentration of 0.2 M KCl in 2 ml. Part (800 μl) of this fraction was incubated with 20 mM Tris-HCl, pH 7.8, 2 mM MgCl_2_, 0.1 mg ml^−1^ BSA, 2 mM DTT, 1 mM CTP and 40 μg of ISPD for 150 min at 37 °C in a final volume of 1 ml. More than 90% of radiolabelled D-ribitol-5-P was converted into [^32^P]-CDP-ribitol in this reaction. Remaining CTP and PPi were destroyed by incubating the reaction with alkaline phosphatase (FastAP, Thermofisher) for 45 min at 37 °C, followed by heat inactivation for 10 min at 75 °C.

*[^3^H]CDP-ribitol synthesis*. 375 nmol of D-ribose-5-P (Sigma) was incubated in a twofold molar excess of [^3^H]-sodium borohydride (specific activity 5 Ci mmol^−1^, Perkin Elmer) for 1 h at RT, brought to pH 5 by addition of HCl, and further processed as described above.

### Cell culture

Human embryonic kidney cells (HEK293), HEK-293-T cells (kind gifts of Jean-Baptiste Demoulin, UCL, Brussels, and Thomas Michiels, UCL, Brussels, respectively), C2C12 myoblast cells (a kind gift of Ormond MacDougald, Ann Arbor) and human skin fibroblasts were cultured in DMEM medium containing 4.5 g l^−1^ D-glucose, 10% fetal calf serum, 2 mM Ultraglutamine I (Lonza), and 100 U ml^−1^ Penicillin/Streptomycin (Lonza). HAP1 cells were obtained from Haplogen and cultured in IMDM medium with the same additives. Fibroblasts were obtained by skin biopsy. Informed consent for genetic analysis was systematically obtained from the parents according to French law. Fibroblasts were treated with 3 mM ribitol for 96 h, changing the medium half-way through this incubation time.

To generate recombinant lentiviruses (for over/re-expression of ISPD, tarI, FKTN, FKRP or α-dystroglycan fragments, as well as knockdown of FGGY), 293 T cells were transiently transfected with lentiviral vectors and second generation packaging plasmids psPAX2 and pMD2.G (kind gifts of Didier Trono, Addgene #12260 and #12259) using the calcium phosphate coprecipitation method as described before^64^,^65^. 24 h to 48 h after transfection, target cells were infected in the presence of 8 μg ml^−1^ polybrene (Sigma). Infected cells were selected for 4 days with 1.5 μg ml^−1^ of puromycin (Thermofisher), 300 μg ml^−1^ of hygromycin (Invivogen) or 1,000 μg ml^−1^ G418 (Invivogen). Empty lentiviral constructs or constructs expressing nonsilencing shRNA (pTRIPZ-negative, GE healthcare) were used to generate control cell lines.

Doxycyclin treatments were performed at 500 ng μl^−1^ where indicated for three days to induce expression of shRNAs (Fig. 4d) or Ispd (Supplementary Figs 8 and 9). Doxycyclin-inducible vectors for Ispd led to some expression of Ispd even in the absence of doxycyclin. This was sufficient to rescue the phenotype of Ispd knockout cell lines. Hence, experiments in Figs 3b and 6b were performed in the absence of doxycycline. Ispd expression in the experiment described in Fig. 4d was achieved by using the construct pOH460, a construct driving constitutive expression of Ispd.

To inactivate genes, we transfected HAP1 cells or HEK293 cells with pairs of CRISPR/Cas9 double-nickase constructs with Lipofectamine 2000 using 0.5 μg of each construct per 12-well plate. Cells were transiently selected with 2 μg ml^−1^ puromycin for 48 h to enrich for transfected cells. Clonal populations were isolated 10 days later, and gene inactivation was verified by nucleotide sequencing of PCR products encompassing the guide RNA target sites.

Differentiation of C2C12 cells into myotubes was induced by replacing fetal bovine serum with 1% horse serum (Life Technologies) when cells reached confluence. Three days later, ribitol was added for 24 h.

### Flow cytometry

α-Dystroglycan glycosylation was assessed by flow cytometry using the IIH6 antibody (Merck Milipore). Cells were washed once with phosphate-buffered saline (PBS) (37 °C) and incubated for 15 min with 1 ml of nonenzymatic cell dissociation solution (Sigma) until they detached. Cells were gently resuspended after addition of 2 ml of PBS and washed twice with 2 ml of PBS, spinning at 500 g for 3 min in between. Fixation was performed for 10 min at RT by resuspending the cells in 2% paraformaldehyde in PBS. In the case of HAP1 cells, this fixation step was left out. Cells were then washed twice with 2 ml of PBS, centrifuging at 1,000 *g* for 3 min in between. For staining, cells were resuspended in 100 μl PBS containing 0.1% FCS and 1:100 IIH6 antibody (Merck Milipore, #05-593), and incubated for 60 min on ice. After washes with PBS containing 0.1% FCS, the cells were stained with 1:100 anti-IgM-FITC antibody (Santa Cruz, sc-2082) for 40 min on ice. Cells were again washed twice and analysed using a Fortessa or FACSverse flow cytometer (BD biosciences).

### Overexpression and purification of recombinant ISPD

Sf9 cells (Life Technologies) were cultured in SF900 III SFM medium (Invitrogen) at 28 °C. For transfection, 10^6^ cells were plated in 1.5 ml of TC100 medium per well of a six-well plate. After one hour at RT, the cells were transfected using 5 μl of Fugene HD (Roche) with 200 ng of flashBAC plasmid and 1 μg of pIG252-1. After overnight incubation, another 0.5 ml of SF900 III medium was added. Virus was harvested 7 days later and serially expanded in Sf9 cells cultured in suspension culture. Titres were determined using a FITC-labelled antibody directed against baculovirus envelope gp64 protein (eBiosciences) and recombinant protein production was initiated by infecting exponentially growing Sf9 cells with a multiplicity of infection of 20. Cells were harvested 48 h later and lysed in Tris-buffered saline containing Triton X-100 (150 mM NaCl, 20 mM Tris-HCl, pH 7.4, 0.2% Triton X-100). After sonication and clarification of the lysate by centrifugation (27,000*g*, 4 °C, 15 min), histidine-tagged ISPD was purified from cell lysates by metal affinity chromatography as described before^64^, and eluted with a gradient of imidazole. Addition of 50 μM CTP was crucial for the stability and activity of the recombinant protein.

### Overexpression and purification of proteins from HEK cells

HEK293 cells were stably infected with vectors driving expression of α-dystroglycan fragments, FKTN or FKRP. Medium was poured off and cells were collected in NETN buffer (150 mM NaCl, 1 mM EDTA, 20 mM Tris-HCl, pH 8.0, 0.2% NP40/Igepal CA-630). Approximately 300 μl of buffer was used for 30 million cells. Lysates were clarified by centrifugation at 27,000 g for 15 min at 4 °C. Per 600 million cells, we added 100 μl of streptavidine Sepharose beads (GE healthcare). When α-dystroglycan was purified from the supernatant of HEK293 cells, we used 100 μl of beads per 40 ml of medium. After rotation for 1 h at 4 °C, beads were washed four times in NETN buffer. For further analysis or enzymatic assays, proteins were either resuspended in 2x the bead volume of NETN or eluted from beads with 0.1% formic acid. Neurofascin was purified as described before^50^.

### CDP-ribitol pyrophosphorylase activity assay

Variable amounts of the indicated phosphate esters and 5.4 ng of recombinant mouse Ispd were incubated in a 20 μl reaction containing 20 mM Tris-HCl, pH 7.8, 20 mM KCl, 2 mM MgCl_2_, 0.1 mg/ml BSA, 2 mM DTT and 1 mM CTP for 30 min at 37 °C. After heat-inactivation for 10 min at 80 °C, 100 μl of water was added, and 110 μl of the sample was analysed by HPLC on a strong anion exchange column (Partisphere SAX, 125 × 4.6 mm) using an Agilent 1100 HPLC system with diode array detector. A gradient of ammonium phosphate, pH 3.7, was applied at a flow rate of 2 ml min^−1^ using 50 mM ammonium phosphate, pH 3.7 (buffer A) and 500 mM ammonium phosphate, pH 3.7 (buffer B), and absorbance at 254 and 280 nm was followed^66^. For the results presented in Fig. 3, the following gradient was used: 0–20 min, 2.5% buffer B; 20–30 min, 2.5–100% buffer B; 30–46 min, 100% buffer B; 47–51 min, 2.5% buffer B. CDP-ribitol eluted at 4.7 min under these conditions. In all other experiments, the gradient was as follows: 0–8 min, 0% buffer B; 8–12 min, 0–5% buffer B; 12–15 min, 5–35% buffer B; 15–20 min, 35–45% buffer B; 20–27 min, 45–50% buffer B; 27–43 min, 50–100% buffer B; 43–48 min, 0% buffer B. CDP-ribitol eluted at 12.7 min under these conditions. In Fig. 1, CDP-ribitol concentrations were determined based on its absorption at 280 nm according to the Lambert–Beer law. In all other experiments, the area of the CDP-ribitol peak was normalized to the total area of all peaks measured at 280 nm (arbitrary units, a.u.).

To assess the identity of CDP-ribitol in biological samples through the reversibility of the ISPD reaction, we incubated 100 μl of neutralized rat muscle extract (see below) in a total volume of 120 μl containing 20 mM Hepes pH 7.4, 2 mM MgCl_2_, 0.1 mg/ml BSA, 2 mM DTT and 30 mM KCl for 30 min at 37 °C, in the presence or absence of recombinant ISPD (3.4 μg ml^−1^) and 0.625 mM NaPPi (where indicated in the figure). The reaction was stopped by heating at 80 °C for 5 min and the mixture was centrifuged for 5 min at 27,000*g* at RT. Subsequently, 100 μl of the supernatant were analysed by HPLC as described above.

### Extraction of metabolites from tissues and cells

Adherent cells were washed once with cold PBS. Ice-cold 5% HClO_4_ (400 μl per 10  cm plate) was added and the cells were scraped. Extracts were spun down for 5 min at 27,000*g* and 4 °C, the supernatant was neutralized with 3M K_2_CO_3_ and centrifuged 10 min at 27,000*g* and 4 °C. HPLC analysis of 100 μl of the supernatant was performed as described above. Muscle or brain tissues were snap-frozen in liquid nitrogen, powdered with a mortar and a pestle, homogenized in three times the volume of ice-cold 5% HClO_4_, and centrifuged for 5 min at 27,000*g* and 4 °C. The supernatant was neutralized with 3 M K_2_CO_3_ and the samples were centrifuged for 10 min at 27,000*g* and 4 °C. The resulting supernatant was either directly analysed by HPLC (see above), or further fractionated for MS analysis. To this end, samples were diluted fourfold, loaded on a Q-sepharose column (20 ml bed volume) and eluted with a NaCl gradient (0–500 mM). Fractions containing CDP-pentitol were identified by HPLC (as described above), pooled and desalted on a Biogel P2 column (40 ml bed volume) equilibrated with water. After concentration by lyophilization, we loaded the sample on a porous graphite column (Thermofisher, Hypercarb, 150 × 2.1 mm). Separation was performed in 0.3% ammonium formate, pH 9.0 using a linear gradient of acetonitrile from 0 to 50% in 20 min at a flow rate of 1 ml min^−1^. Fractions containing CDP-ribitol were identified by HPLC and the solvent was evaporated by lyophilization. Samples were resuspended in 60% acetonitrile and analysed by direct injection into a LTQ- or Orbitrap XL mass spectrometer (Thermo Scientific), the latter giving access to the exact masses of the analytes.

Briefly, the MS was calibrated for mass accuracy in negative mode before analysing the samples using an ESI ionization source operated in negative mode. The source voltage and current were set at 5 kV and 70 μA, respectively, while the capillary voltage was set at −40 V. Full MS scans between 450 and 650 *m/z* were performed. For the MS/MS analysis, the ions of interest were fragmented by CID with 20% collisional energy and the resulting spectra were recorded.

### Incorporation of [^32^P]-CDP-ribitol into α-dystroglycan

Incorporation of radioactive D-ribitol-5-P in α-dystroglycan was obtained by incubating FKTN or FKRP with an approximately 10-fold excess of purified α-dystroglycan bound to streptavidin beads in 20 mM MES, pH 6.5, 50 μg/ml BSA, 2 mM DTT, 0.5% Triton X-100, 2 mM MnCl_2_ overnight at 30 °C. To remove unincorporated nucleotides and BSA, the streptavidin beads were washed 1 × (before SDS-PAGE) or three times (before scintillation counting) with 1 ml of NETN buffer. Samples were incubated with an equal volume of SB 2 × buffer (120 mM Tris-HCl pH 7.4, 16% glycerol, 4% SDS, 2% (v/v) mercaptoethanol, 0.02% (m/v) bromophenol blue), heated up at 37 °C for 10 min and then loaded on 10% Bis-tris pH 7.0 gels, run at 200 V in MOPS–SDS buffer for 25 min. Radioactivity was detected in the gels with a storage phosphorimager (Typhoon TRIO, GE Healthcare), and protein was visualized by Coomassie Blue or silver staining. Scintillation counting was performed with Ultima Gold scintillation fluid (Perkin Elmer) and a Tricarb 2800 TR scintillation counter (Perkin Elmer).

For treatment of ribitol-phosphorylated α-dystroglycan with alkaline phosphatase and glycosidases, samples were incubated as described above with [^32^P]-CDP-ribitol. After incubation, samples were washed three times with NETN, eluted from the beads with 0.1% formic acid, spinned down to eliminate the beads, neutralized with 0.3% ammonium formate pH 9.0 and distributed over several tubes to be dried down in a SpeedVac vacuum concentrator (Thermo Scientific). For alkaline phosphatase treatment, samples were incubated with 20 U of calf intestine alkaline phosphatase or not (control) for 1 h at 37 °C in the buffer supplied by the manufacturer (Roche). To test the effect of glycosidases, ribitol-phosphorylated samples were denatured and incubated in G7 buffer with or without deglycosylation enzyme mix (BioLabs) overnight at 37 °C as recommended by the manufacturer. Samples were then analysed by SDS–PAGE followed by storage phosphorimager analysis or silver staining. Where indicated, purified α-dystroglycan was incubated overnight in G7 buffer with or without deglycosylation enzyme mix (BioLabs) followed by ribitol-phosphorylation with FKTN and FKRP. In this case, the initial denaturation step was omitted.

### GC/MS analysis of monosaccharides

Monosaccharide (for the sake of simplicity, we include ribitol in the ‘monosaccharide' designation) composition was determined by GC/MS analysis of the TMS monosaccharides. For data represented in Supplementary Figs 8 and 9, the dried glycoprotein was incubated for 16 h at 70 °C under argon with 100 μl methanolic HCl, re-N-acetylated for 30 min at RT (50 μl methanol, 5 μl pyridine and 5 μl acetic anhydride), derivatized with N-methyl-N-trimethylsilyl-trifluoracetamide (Macherey-Nagel) during 10 min at 70 °C and analysed on a Fison MD800 GC/MS (Thermon Electron) with a 15 m RTX5-SILMS column (Restek). After an initial temperature of 100 °C for 1 min, a gradient of 6 °C min^−1^ up to 260 °C was used. MS spectra were obtained by electron impact ionization. Masses between *m/z* 100 and 700 were scanned every second at 400 V. Chromatograms and spectra were analysed with the software MassLynx. For data represented in Fig. 6, monosaccharides were derivatized with N-methyl-N-trimethylsilyl-trifluoracetamide (Macherey-Nagel) during 30 min at 37 °C and analysed with an Agilent 7890A GC equipped with a 30 m DB-5 ultra inert capillary column connected to an Agilent 5977 mass sensitive detector operating under electron impact ionization at 70 eV. One microliter of sample was injected in splitless mode at 270 °C, using helium as the carrier gas at a flow rate of 1 ml min^−1^. The GC oven temperature was held at 100 °C for 3 min and increased to 300 °C at 3.5 °C min^−1^. The MS source and quadrupole detector were held at 230 °C and 150 °C, respectively. Data were acquired in combined selected-ion monitoring and scanning mode (68–700 *m/z*). Selected-ion monitoring chromatograms were integrated using Masshunter software (Agilent) and areas were normalized to the abundance of methyl-glycine (‘relative ribitol incorporation'), a side-product formed due to partial hydrolysis of the protein component of α-dystroglycan. Within each experiment, ribitol levels were normalized to the abundance observed in wild-type cells or wild-type cells expressing a negative control plasmid.

### Laminin overlay assay and western blot

Laminin overlay assays were performed as described before with slight modifications^2^. Cells were lysed in PBS containing 1% Triton X-100 and Complete proteinase inhibitor cocktail (Roche). After brief sonication, samples were centrifuged 15 min at 27,000*g* and 4 °C. Supernatants were diluted 1:1 in PBS and incubated under gentle rotation for 16 h at 4 °C with WGA-Agarose beads (Vector Laboratories) using 100 μl of beads per 900 μg (fibroblasts) or 1,400 μg (HAP1 cells) of protein. Beads were washed 3x with 1 ml of PBS containing 0.1% Triton X-100, proteins were released by incubation for 10 min at 72 °C in the presence of reducing sample buffer and resolved on 3-8% Tris-acetate gels (Life Technologies) for 75 min at 130 V. After overnight transfer onto PVDF membrane, membranes were blocked with laminin-binding buffer (LBB, 50 mM Tris-HCl pH 7.4, 150 mM NaCl, 1 mM MgCl_2_ and 1 mM CaCl_2_) containing 3% BSA (Sigma) for 1 h at RT and incubated overnight with 1.15 μg ml^−1^ Laminin-111 (Sigma, L2020) in LBB. After three 10 min washes with LBB, membranes were incubated for 2 h at RT with rabbit anti-laminin antibody (Sigma, L9393) diluted 1:1,000 in LBB containing 3% BSA, washed another three times for 10 min with LBB, incubated for 1 h at RT with horse-radish-peroxidase-coupled donkey anti-rabbit IgG antibody (GE healthcare, NA934V) diluted 1:15,000 in LBB containing 3% BSA, followed by three more 10 min washes in LBB at RT. Chemiluminescent signals were detected using a home-made chemiluminescent detection system^67^. In parallel to the laminin overlay, part of the membranes were incubated with anti-β-dystroglycan antibody (Santa Cruz, clone 7D11, sc-33701, 1:1,000) using a conventional western blot protocol as described before^64^. Western blot analysis for FKTN (Abcam, clone id EPR7913, #5982-1, 1:1,000) and FLAG-tag (Sigma F1804, 1:1,000) was performed likewise. Uncropped images of the laminin overlay assay and western blots can be seen in Supplementary Fig. 14.

### Mice

Three-week-old C57BL6 mice were fed with normal chow plus 2% ribitol in the drinking water for three weeks. After euthanasia, muscle and brain were dissected and snap-frozen before further analysis (see above). To study the effect of Sorbinil on CDP-ribitol levels, 2-month-old male CD1 mice were injected intraperitoneally 25 mg kg^−1^ per day Sorbinil for 6 days. After euthanasia, muscle and brain tissue were snap-frozen on liquid nitrogen and ground-up with a mortar and pestle on liquid nitrogen. About 200 mg of tissue were homogenized in three volumes of 0.5 M HClO_4_, and processed as described for tissue culture samples above. Before HPLC analysis, samples were digested with alkaline phosphatase to remove nucleotides that partially overlap with the CDP-ribitol peak. HPLC was performed as described above, but peak areas observed at 280 nm were integrated and normalized to the total area of all peaks observed at this wavelength.

Animal experiments were approved by the Université catholique de Louvain local ethics committee and conducted in accordance with the guidelines of the European Convention for the Protection of Vertebrate Animals used for Experimental and Other Scientific Purposes.

### Statistical evaluation

For simple comparisons, two-tailed *t*-tests were performed in Microsoft Excel. When multiple comparisons were performed, analysis of variance was followed by Dunnett's test for multiple comparisons performed in Graphpad Prism.

### Data availability

The authors declare that all data supporting the findings of this study are available within the article and its Supplementary Information files.

## Additional information

**How to cite this article:** Gerin, I. *et al*. ISPD produces CDP-ribitol used by FKTN and FKRP to transfer ribitol phosphate onto α-dystroglycan. *Nat. Commun.* 7:11534 doi: 10.1038/ncomms11534 (2016).

## Supplementary Material

Supplementary Information Supplementary Figures 1-14, Supplementary Tables 1-2 and Supplementary References.
