# Supplementary material for: ISPD produces CDP-ribitol used by FKTN and FKRP to transfer ribitol phosphate onto α-dystroglycan
Source: Nat Commun. 2016 May 19;7:11534. doi: 10.1038/ncomms11534 (PMC4873967; doi:10.1038/ncomms11534)
Supplement: Supplementary Information — Supplementary Figures 1-14, Supplementary Tables 1-2 and Supplementary References. [file ncomms11534-s1.pdf]

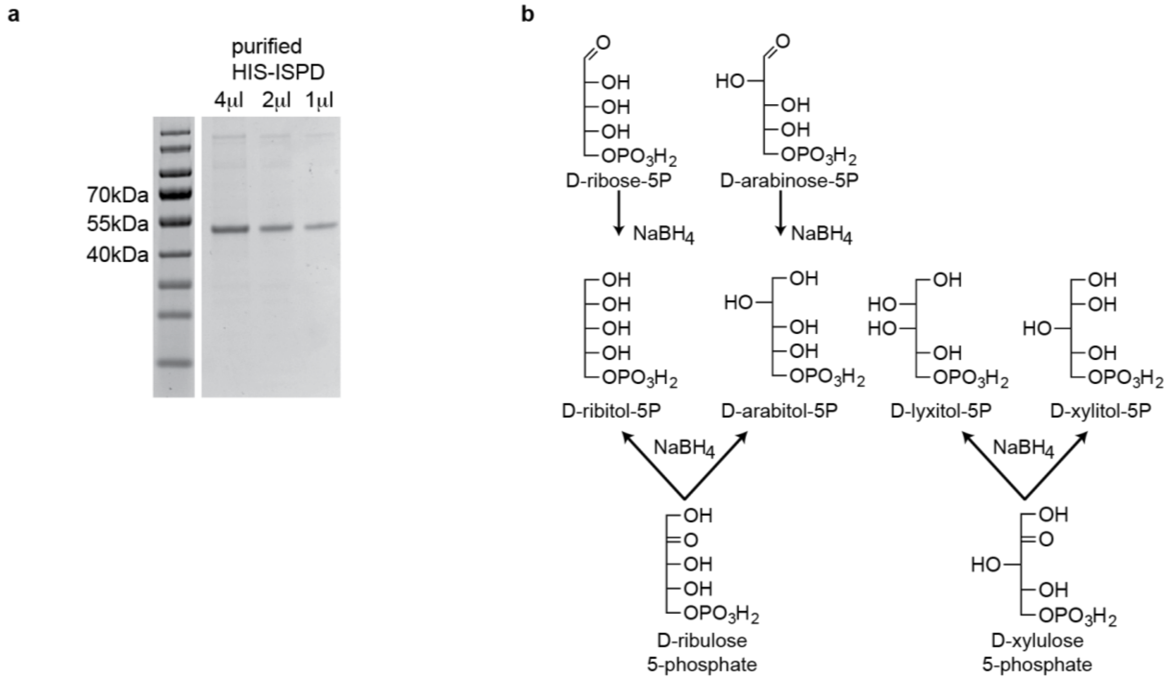

Supplementary Figure 1. Characterization of recombinant mouse Ispd and its substrates.
a, Purified mouse Ispd with an N-terminal hexahistidine tag was separated by SDS-PAGE and stained with Coomassie Blue.
b, Generation of pentitol-phosphates by reduction of pentose-phosphates or pentulose-phosphates by sodium borohydride.

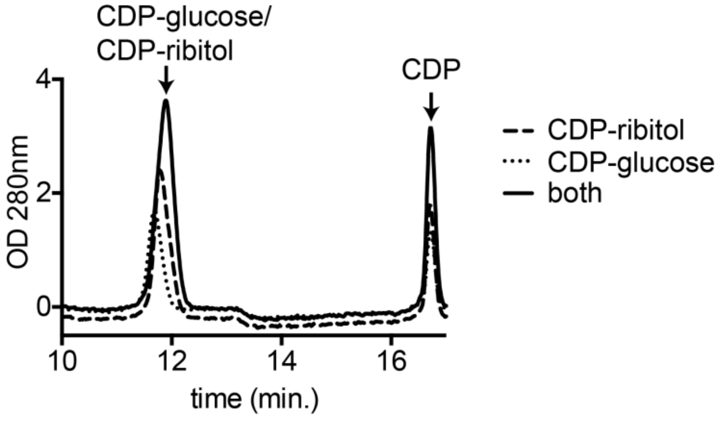

Supplementary Figure 2. CDP-glucose coelutes with CDP-ribitol in HPLC analysis.
HPLC analysis of CDP-glucose (synthesized using UDP-glucose pyrophosphorylase, CTP and glucose-1P), CDP-ribitol (synthesized using recombinant mouse Ispd, CTP and D-ribitol-5P) and a mixture of CDP-glucose and CDP-ribitol.

Clone B3 (#1): 23 bp deletion

```
Seq_1  241   GGCAGCTGTGTTGCCTGCCGGGGGGTGCGGGGAGAGGATGGGGGTCCCCACCCCGAAGCA  300
             ||||||||||||||||||||||||||||||||||||||||||||||||||||
Seq_2  216   GGCAGCTGTGTTGCCTGCCGGGGGGTGCGGGGAGAGGATGGGGGTCCCCACC--------  267

Seq_1  301   ATTCTGCCCCATCCTGGAGAGGCCGCTCATCAGCTACACCCTACAGGCCCTGGAGAGgta  360
                            |||||||||||||||||||||||||||||||||||||||||||||
Seq_2  268   ---------------GGAGAGGCCGCTCATCAGCTACACCCTACAGGCCCTGGAGAGGTA  312
```

Clone D12 (#2): 61 bp deletion

```
Seq_1  240   TGGCAGCTGTGTTGCCTGCCGGGGGGTGCGGGGAGAGGATGGGGGTCCCCACCCCGAAGC  299
             ||||||||||||||||||||||||||||||||||||||||||||||||||
Seq_2  210   TGGCAGCTGTGTTGCCTGCCGGGGGGTGCGGGGAGAGGATGGGGGTCCCC----------  259

Seq_1  300   AATTCTGCCCCATCCTGGAGAGGCCGCTCATCAGCTACACCCTACAGGCCCTGGAGAGgt  359
                                                                |||||||||
Seq_2  260   ---------------------------------------------------TGGAGAGGT  268
```

Clone F5 (#3): 1 nucleotide deletion

```
Seq_1  241   GGCAGCTGTGTTGCCTGCCGGGGGGTGCGGGGAGAGGATGGGGGTCCCCACCCCGAAGCA  300
             |||||||||||||||||||||||||||||||||||||||||||||||||||  |||||||
Seq_2  216   GGCAGCTGTGTTGCCTGCCGGGGGGTGCGGGGAGAGGATGGGGGTCCCCACA-CGAAGCA  274

Seq_1  301   ATTCTGCCCCATCCTGGAGAGGCCGCTCATCAGCTACACCCTACAGGCCCTGGAGAGgta  360
             ||||||||||||||||||||||||||||||||||||||||||||||||||||||||||||
Seq_2  275   ATTCTGCCCCATCCTGGAGAGGCCGCTCATCAGCTACACCCTACAGGCCCTGGAGAGGTA  334
```

Supplementary Figure 3. ISPD mutations in HAP1 cells induced by CRISPR/Cas9 double-nickase approach.
The three selected clones contain deletions in exon 1 of ISPD that lead to frameshifts. Locations of the guide RNAs are underlined.

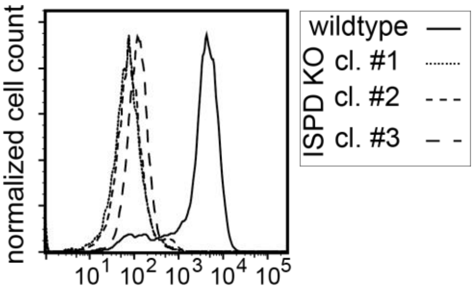

Supplementary Figure 4. ISPD inactivation in HAP1 cells leads to reduced dystroglycan glycosylation.
Glycosylation status of HAP1 parental cells and three independent knockout clones was assessed by flow cytometry using the antibody IIH6.

Clone B8 (#1): 49 bp insertion

```
Seq_1  598   TGCAGACCAGCCAATTAAGAATTGGGAGCCCCAGTTCAA---------------------  636
             ||||||||||||||||||||| |||||||||||||||||
Seq_2  92    TGCAGACCAGCCAATTAAGAAGTGGGAGCCCCAGTTCAAGAGCAGTCCTCCGAGGACATC  151


Seq_1  637   ----------------------------CCACCATGAGCAGTCCTCCGAGGACATCTGGG  668
                                         ||||||||||||||||||||||||||||||||
Seq_2  152   TGGGCTGCGTGCTGTGTTGTCACAAAGGCCACCATGAGCAGTCCTCCGAGGACATCTGGG  211
```

Clone B11 (#2) : 17 bp deletion

```
Seq_1  597   TTGCAGACCAGCCAATTAAGAATTGGGAGCCCCAGTTCAACCACCATGAGCAGTCCTCCG  656
             |||||||||||||||||||||| |||||||||||||||                 |||||
Seq_2  95    TTGCAGACCAGCCAATTAAGAAGTGGGAGCCCCAGTTC-----------------CTCCG  137


Seq_1  657   AGGACATCTGGGCTGCGTGCTGTGTTGTCACAAAGgtatgggcaaaactggtgttctctc  716
             ||||||||||||||||||||||||||||||||||||||||||||||||||||||||||||
Seq_2  138   AGGACATCTGGGCTGCGTGCTGTGTTGTCACAAAGGTATGGGCAAAACTGGTGTTCTCTC  197
```

Clone D3 (#3): 26bp deletion

```
Seq_1  597   TTGCAGACCAGCCAATTAAGAATTGGGAGCCCCAGTTCAACCACCATGAGCAGTCCTCCG  656
             |||||||||||||||||||||| ||||||                          |||||
Seq_2  95    TTGCAGACCAGCCAATTAAGAAGTGGGAG--------------------------CTCCG  128


Seq_1  657   AGGACATCTGGGCTGCGTGCTGTGTTGTCACAAAGgtatgggcaaaactggtgttctctc  716
             ||||||||||||||||||||||||||||||||||||||||||||||||||||||||||||
Seq_2  129   AGGACATCTGGGCTGCGTGCTGTGTTGTCACAAAGGTATGGGCAAAACTGGTGTTCTCTC  188
```

Supplementary Figure 5. FGGY mutations in HAP1 cells induced by CRISPR/Cas9 double-nickase approach.
The three selected clones contain deletions or insertions in exon 1 of FGGY that lead to frameshifts. Locations of the guide RNAs are underlined.

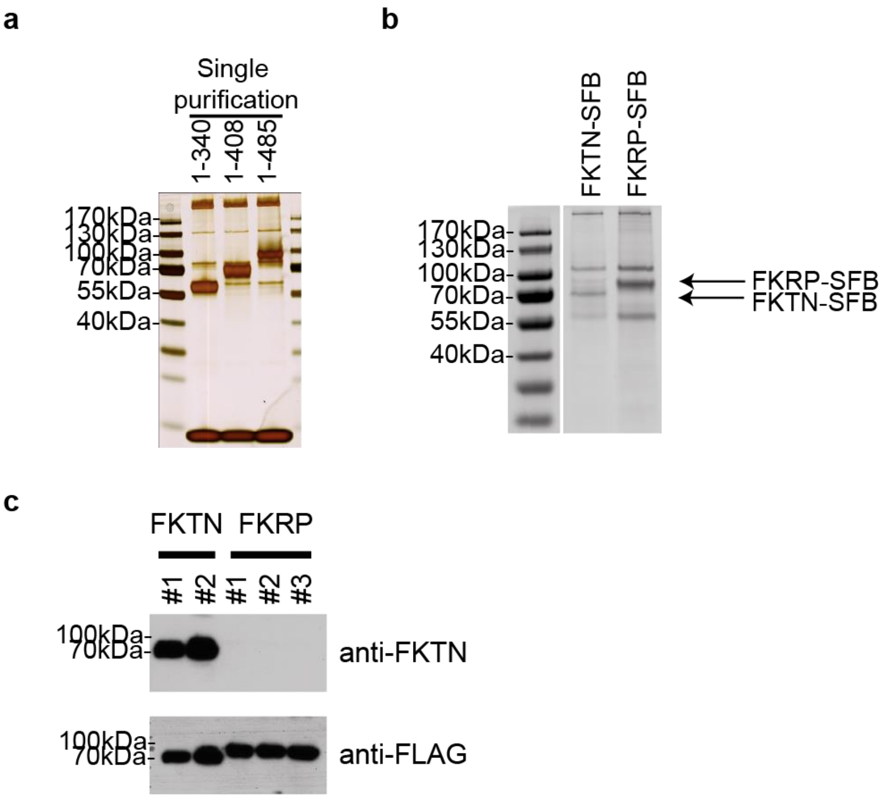

Supplementary Figure 6. Purification of FKTN, FKRP and rabbit α-dystroglycan by affinity purification.

a, Fusion proteins of N-terminal fragments of rabbit α-dystroglycan with a C-terminal SFB-tag were purified in a single step with Sepharose beads covalently coupled to streptavidin (lanes 2-4), were resolved by SDS-PAGE and are visualized by silver staining.

b, Fusion proteins comprising full-length mouse Fktn and Fkrp with a C-terminal SFB-tag were partially purified with Sepharose beads covalently coupled to streptavidin, resolved by SDS-PAGE and stained with Coomassie Blue.

c, Affinity-purified FKTN and FKRP containing a C-terminal SFB-tag were analyzed by western blot using an antibody directed against FKTN and the FLAG epitope tag.

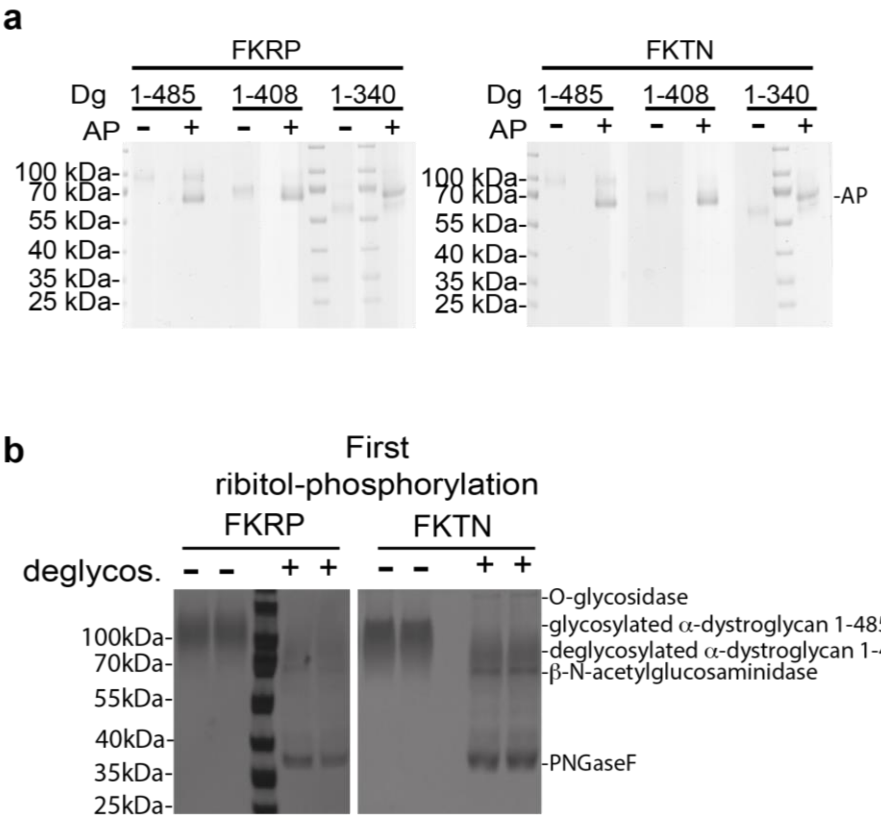

Supplementary Figure 7. SDS-PAGE analysis and silver-staining/Coomassie Blue staining of samples used in ribitol-phosphorylation experiments.

a, Coomassie Blue staining of the gel used to obtain the ^{32}P-signal shown in Figure 5. The position of the alkaline phosphatase (AP) protein is indicated.

b, Silver staining of a parallel gel analysing the samples used to obtain the ^{32}P signal shown in Figure 5d (left two panels). The samples shown in the two right panels of Figure 5d contain 10 µg BSA, which largely obscures other proteins of interest. Hence the corresponding gel is not shown.

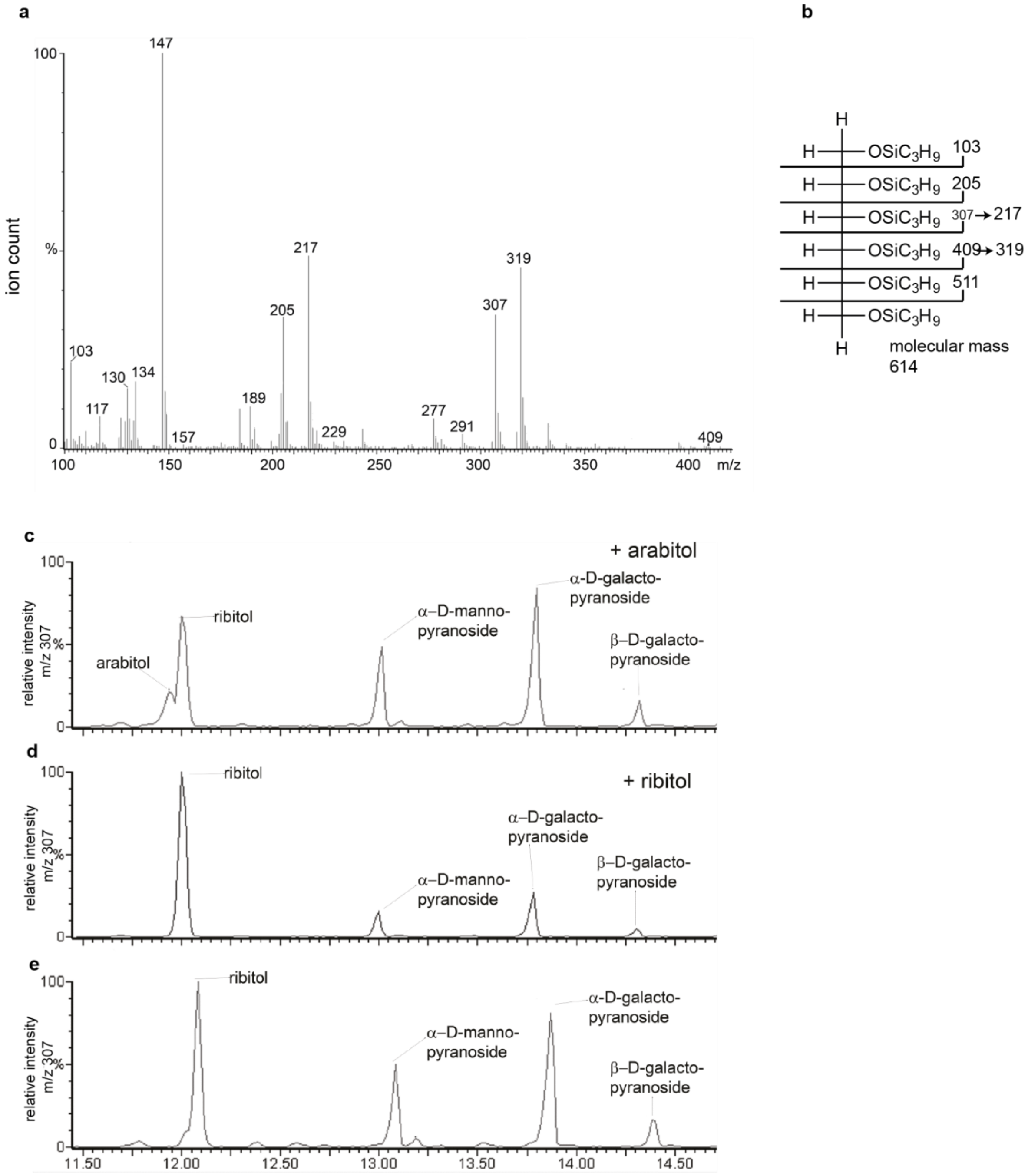

Supplementary Figure 8. Identification of ribitol bound to α-dystroglycan.
a&b, HEK293-cells engineered to express an α-dystroglycan fragment (comprising amino acids 1-485 and a C-terminal SFB tag) and ISPD under the control of a doxycycline-regulated promoter were incubated with doxycycline for four days. α-dystroglycan was purified by affinity chromatography from the cell supernatant**.** N- and O-glycans were released from α-dystroglycan and depolymerized by treatment with methanolic HCl before re-N-acetylation and derivatisation with TMS. The EI spectrum obtained for the peak at 12.084 min (**a**) shows a fragment pattern of a pentitol (**b**) and its retention time is consistent with ribitol. The fragments with an m/z of 512, 409, 307, 205 and 103 represent fragments that have lost one, two, three or four carbon units as indicated in the schematic (**b**).
c-e, Arabitol (**c**) and ribitol (**d**) were added to hydrolyzed dystroglycan glycans (**e**) before derivatization in order to show that the observed pentitol coelutes with ribitol.

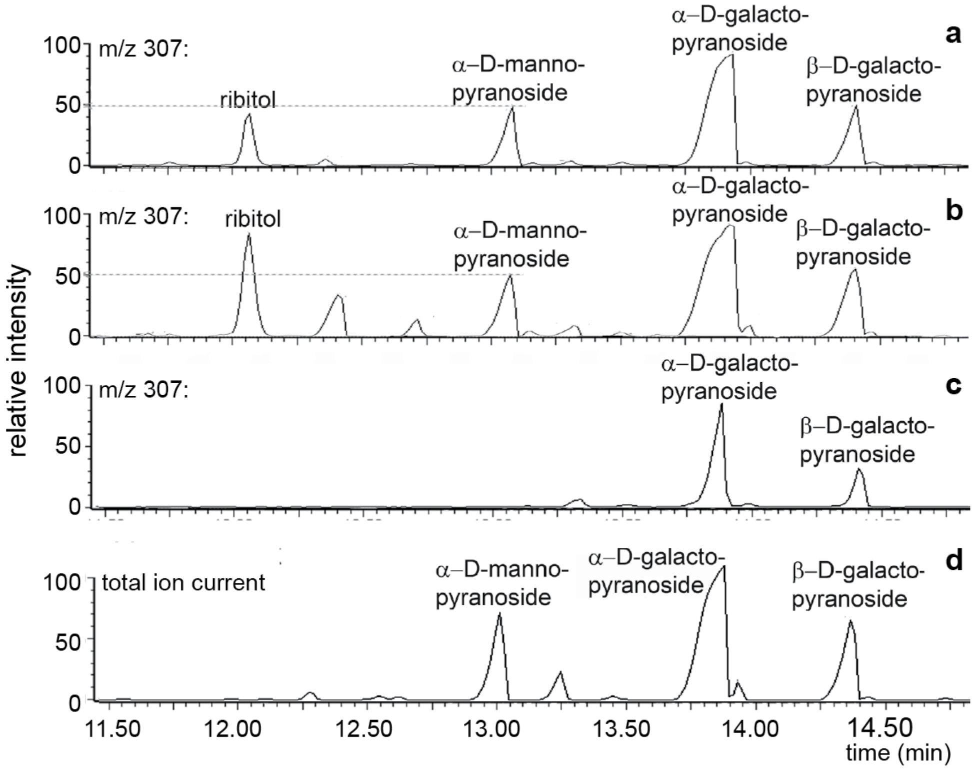

Supplementary Figure 9. Incorporation of ribitol in α-dystroglycan but not in neurofascin, another O-glycoprotein.

a-b, HEK293-cells engineered to express an α-dystroglycan fragment (comprising amino acids 1-485 and a C-terminal SFB tag) and ISPD under the control of a doxycycline-regulated promoter were incubated without **(a)** or with **(b)** doxycycline. α-dystroglycan was purified by affinity chromatography from the cell supernatant. N- and O-glycans were released from α-dystroglycan and depolymerized by treatment with methanolic HCl before re-N-acetylation, derivatisation with TMS and analysis by GC-MS. Intensity for the ion at m/z 307 are shown here.

c, GC-MS analysis of monosaccharides similarly isolated from neurofascin, an O-glycoprotein recombinantly expressed in HEK293 cells [1].

d, Total ion current of the sample presented in panel (**a**), revealing the relative amount of ribitol in comparison to the other hexoses.

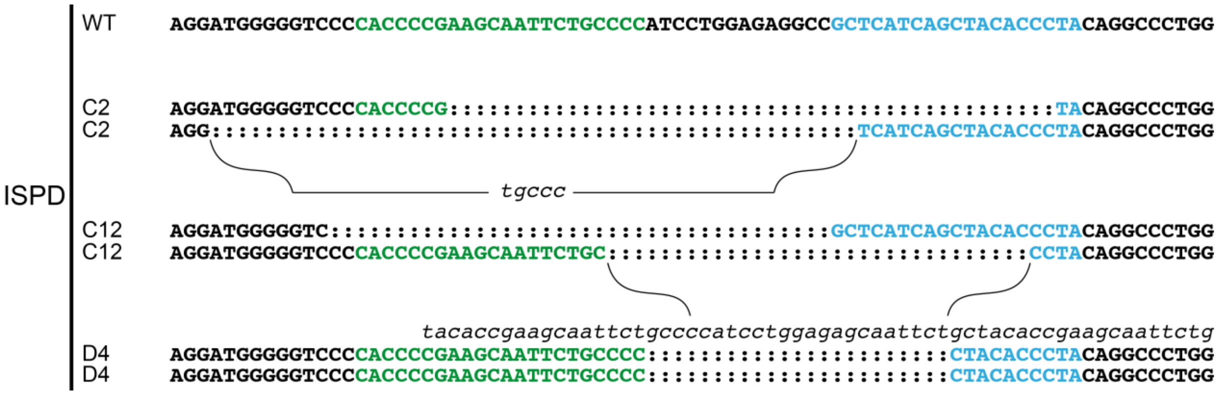

Supplementary Figure 10. ISPD mutations in HEK293 cells induced by CRISPR/Cas9 double-nickase.
The three selected clones contain deletions that lead to frameshifts. Locations of the guide RNAs are highlighted in color. Clones C12, C2 and D4 correspond to clones #1, #2 and #3 in Figure 6, respectively.

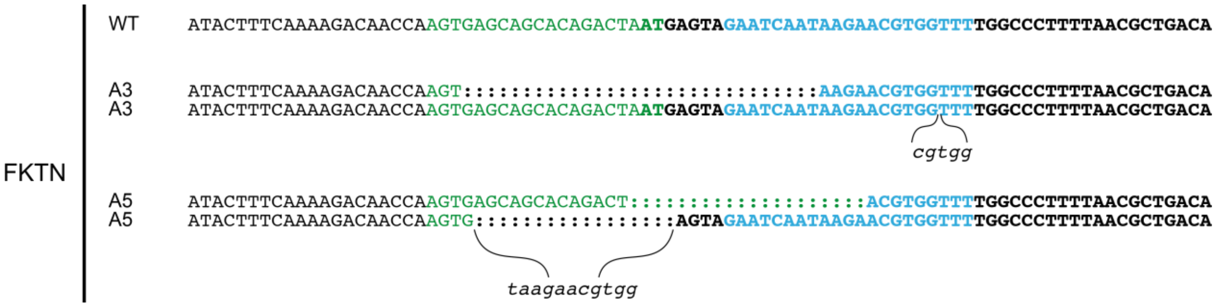

Supplementary Figure 11. FKTN mutations in HEK293 cells induced by CRISPR/Cas9 double-nickase.
The three selected clones contain deletions that lead to frameshifts. Locations of the guide RNAs are highlighted in color. Clone A3 and A5 correspond to clones #1 and #2 in Figure 6, respectively.

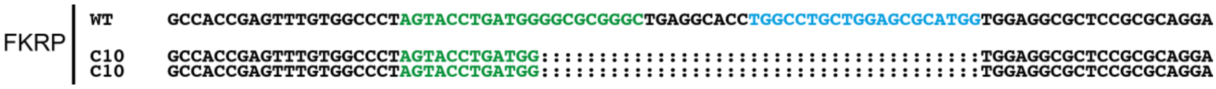

Supplementary Figure 12. FKRP mutations in HEK293 cells induced by CRISPR/Cas9 double-nickase.
The clones contain deletions that lead to a frameshift. Locations of the guide RNAs are highlighted in color.

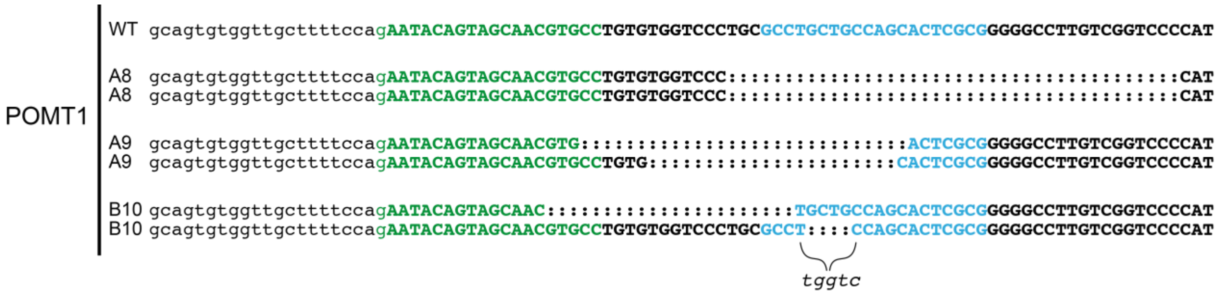

Supplementary Figure 13. POMT1 mutations in HEK293 cells induced by CRISPR/Cas9 double-nickase.
The three selected clones contain deletions that lead to frameshifts. Locations of the guide RNAs are highlighted in color. Clones A8, A9 and B10 correspond to clones #1, #2 and #3, respectively.

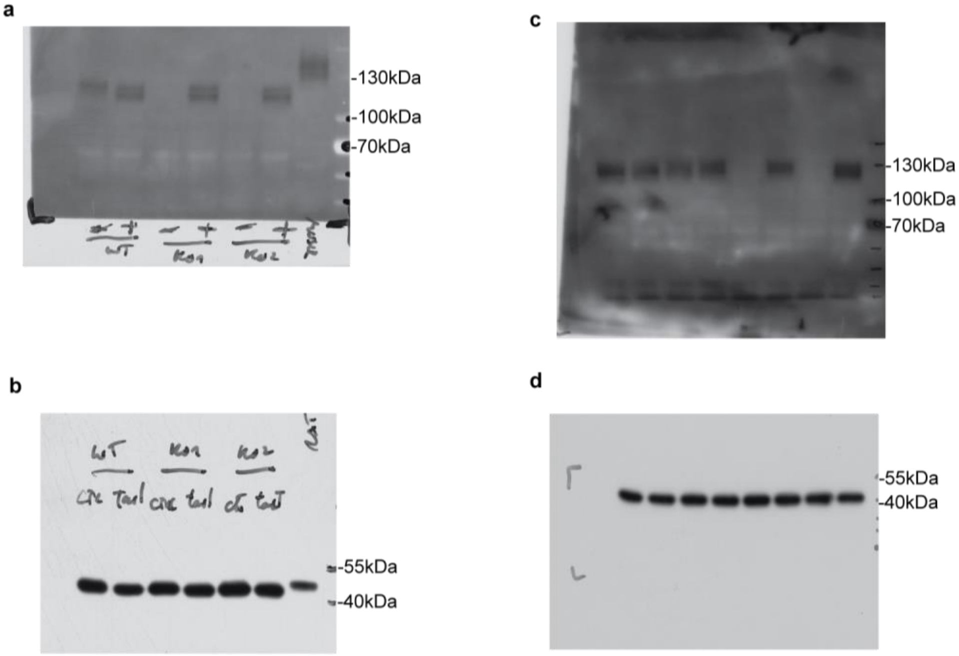

Supplementary Figure 14. Uncropped Western Blots and laminin overlay.
a&b, Uncropped images corresponding to laminin overlay (**a**) and β-dystroglycan western blot (**b**) presented in Fig. 3e. The band on the right corresponds to rat muscle extracts.
c&d, Uncropped images corresponding to laminin overlay (**c**) and β-dystroglycan western blot (**d**) presented in Fig. 7e. Both blots were run in parallel with the same samples.

**Tested substrates with no activity:**
- ATP, UTP
- glycerol-3-P, glycerol-2-P,<br>- 2-P-glycerate, 3-P-glycerate, 2,3-BP-glycerate<br>- 2-C-methyl-D-erythritol-4-P<br>- fructose-6-P, glucose-6-P, arabinose-5-P<br>- glucose-1-P, fructose-1-P<br>- inositol-1-P, inositol-2-P<br>- sorbitol-6-P, mannitol-1-P, erythritol-4-P<br>- fructose-1,6-BP, glucose-1,6-BP
**Tested substrates with <10% of the activity on ribitol-5P at 1 mM**
- Allitol-6-P, arabitol-5-P<br>- D-ribose-5-P, D-ribulose-5-P, D-xylulose-5-P.

Supplementary Table 1: Compounds evaluated as substrates for ISPD at 1 mM.

Patient	Allele 1	Allele 2	Phenotype
**ISPD #1**	c.54-55delGAinsTGC<br>(p.Ser19AlafsX97)	c.677A>G<br>(p.Tyr226Cys)	CMD; no mental retardation
**ISPD #2**	c.554C>T<br>(p.Pro185Leu)	c.1044dup<br>(p.Gln349Serfs*11)	CMD; no mental retardation
**ISPD #3**	c.802C>T<br>(p.Arg268*)	c.1114_1116delGTT<br>(p.Val372del)	CMD; no mental retardation
**ISPD #4**	c.367G>A<br>(p.Gly123Arg)	Exon 3 deletion	CMD; CNS symptoms unknown
**POMT1**	c.2080C>T<br>(p.Q694X)	c.2143insA<br>(p.T715fs>730X)	Type II lissencephaly

Supplementary Table 2: Genotypes and clinical phenotypes of patients.
CMD, congenital muscle dystrophy; CNS, central nervous system.

References:

1. Pacharra, S., Hanisch, F.G. & Breloy, I. Neurofascin 186 is O-mannosylated within and outside of the mucin domain. *Journal of proteome research* **11**, 3955-3964 (2012).
